# DDR2-COL11A1 Transcriptional Coupling as a Candidate Therapeutic Target in Colorectal Cancer: Integrative Transcriptomic and Deep Learning Validation

**DOI:** 10.3390/ijms27052509

**Published:** 2026-03-09

**Authors:** Yasemin Başbınar, Ömer Akgüller, Asım Leblebici, Gizem Çalıbaşı Koçal, Mehmet Ali Balcı, Zerrin Isik, Hülya Ellidokuz

**Affiliations:** 1 Department of Translational Oncology, Oncology Institute, Dokuz Eylul University, Izmir 35340, Turkey; gizem.calibasi@deu.edu.tr; 2 Department of Mathematics, Faculty of Science, Mugla Sitki Kocman University, Mugla 48000, Turkey; oakguller@mu.edu.tr; 3 Oncology Department, Institute of Health Sciences, Dokuz Eylul University, Izmir 35340, Turkey; 4 Department of Information Technologies, Izmir Institute of Technology, Izmir 35430, Turkey; asimleblebici@iyte.edu.tr; 5 Department of Computer Science, Faculty of Science, Vrije Universiteit Amsterdam, 1081 HV Amsterdam, The Netherlands; z.isik@vu.nl; 6 Department of Preventive Oncology, Oncology Institute, Dokuz Eylul University, Izmir 35340, Turkey; hulya.ellidokuz@deu.edu.tr

**Keywords:** discoidin domain receptor, colorectal cancer, transcriptional coupling, extracellular matrix remodeling, deep learning, coexpression analysis, matrix metalloproteinases

## Abstract

Extracellular matrix (ECM) remodeling is a hallmark of colorectal cancer progression, yet the transcriptional mechanisms coordinating collagen deposition and matrix metalloproteinase activation remain incompletely understood. We performed integrated computational analysis of 680 samples across normal mucosa, adenoma, and carcinoma stages to characterize discoidin domain receptor (DDR)-mediated transcriptional networks during tumorigenesis. Stage-stratified correlation analysis of fourteen pathway genes revealed profound divergence between DDR1 and DDR2; DDR1 correlations remained weak across all stages, while DDR2 correlations strengthened 2.59-fold from normal to carcinoma. DDR2-COL11A1 exhibited the most dramatic coupling intensification, increasing from $R^{2}=0.007$ in normal tissue to $R^{2}=0.549$ in carcinoma, accompanied by 1.99-fold COL11A1 upregulation. Remarkably, pathway activation occurred despite stable DDR2 expression, indicating enhanced transcriptional coupling efficiency rather than receptor upregulation as the primary mechanism. Deep neural network classification achieved 93.14% accuracy distinguishing disease stages, with SHAP analysis independently validating DDR2-COL11A1 as the most important gene interaction for cancer classification. These findings establish DDR2-specific transcriptional coupling as a functionally important mechanism in colorectal cancer progression and identify COL11A1 as a critical downstream target, suggesting novel therapeutic strategies targeting coupling efficiency rather than receptor abundance.

## 1. Introduction

Colorectal cancer remains the third most commonly diagnosed malignancy and the second leading cause of cancer-related mortality worldwide, accounting for approximately 1.9 million new cases and 935,000 deaths annually [1,2,3]. Despite advances in screening, surgical techniques, and adjuvant chemotherapy, five-year survival rates for advanced-stage disease remain below 15%, highlighting the urgent need for novel therapeutic strategies targeting the molecular mechanisms underlying tumor progression and metastasis [4,5,6]. The transition from benign adenomatous polyps through increasingly dysplastic intermediate stages to invasive adenocarcinoma represents a well-characterized multi-step progression sequence, yet the transcriptional programs coordinating this evolution, particularly those governing extracellular matrix remodeling, remain incompletely understood [7,8,9].

Extracellular matrix remodeling has emerged as a critical hallmark of cancer progression, enabling tumor cells to escape their primary niche, invade surrounding tissues, intravasate into circulation, and establish distant metastatic deposits [10,11]. The tumor microenvironment undergoes dramatic architectural reorganization during colorectal tumorigenesis, characterized by excessive deposition of fibrillar collagens, altered collagen crosslinking patterns, increased matrix stiffness, and enhanced proteolytic degradation by matrix metalloproteinases [12,13,14]. These changes create a biomechanically permissive environment that facilitates tumor cell migration while simultaneously promoting cancer-associated fibroblast activation, angiogenesis, and immune evasion. Elevated collagen deposition, particularly of fibrillar collagens including types I, III, and XI, is correlated with poor prognosis across multiple cancer types and has been proposed as both a prognostic biomarker and therapeutic target [15,16].

The discoidin domain receptor (DDR) family of receptor tyrosine kinases, comprising DDR1 and DDR2, represents the only known class of receptors activated by collagen binding rather than soluble ligands, positioning them as critical sensors of ECM composition and mechanical properties [17,18,19,20]. DDR1 and DDR2 share structural similarity, each containing an extracellular discoidin homology domain that binds various collagen types, a transmembrane domain, a large intracellular juxtamembrane region, and a conserved tyrosine kinase domain. Despite this structural conservation, accumulating evidence suggests functional divergence between the two receptors. DDR1 is predominantly expressed in epithelial cells and has been implicated in cell adhesion, epithelial–mesenchymal transition, and collective cell migration [21,22]. DDR2 shows broader expression across mesenchymal cells, fibroblasts, and certain cancer cell types, and has been linked to collagen remodeling, matrix metalloproteinase expression, and invasive behavior [17,23].

Activation of DDR receptors by collagen binding triggers autophosphorylation and recruitment of downstream signaling effectors, initiating cascades including Ras-MAPK, PI3K-Akt, Src family kinases, and JAK-STAT pathways that ultimately regulate gene expression programs controlling proliferation, survival, migration, and matrix remodeling [24,25]. Unlike most receptor tyrosine kinases, which exhibit rapid activation kinetics measured in minutes, activation of DDR is remarkably slow, with phosphorylation peaking hours after collagen stimulation and persisting for extended periods. This suggests a role in sustained transcriptional regulation rather than acute signaling responses [26,27]. Their unique temporal profile positions DDRs as potential transcriptional coordinators of long-term matrix remodeling programs during cancer progression.

The functional relationship between DDR activation and matrix metalloproteinase (MMP) expression has been documented in multiple contexts. DDR2 activation in fibroblasts and cancer cells promotes transcriptional upregulation of several MMP family members, including MMP1, MMP2, and MMP13, through activation of transcription factors including AP-1, NF-*κ*B, and Runx2 [28,29,30]. Similarly, DDR signaling has been implicated in regulating collagen gene expression, creating potential feedforward loops wherein collagen-activated DDRs drive expression of both matrix degradation enzymes and additional collagen isoforms [31,32]. However, the existence and functional importance of coordinated transcriptional coupling between DDR receptors and their downstream matrix remodeling targets during cancer progression has not been systematically characterized at the pathway level.

Mounting evidence implicates DDR2 specifically in colorectal cancer pathogenesis. Elevated DDR2 expression has been reported in colorectal cancer tissues compared to normal mucosa, and high DDR2 levels correlate with advanced stage, lymph node metastasis, and shortened overall survival in some patient cohorts [33,34]. Functional studies using cell lines and xenograft models demonstrate that DDR2 knockdown or pharmacological inhibition reduces colorectal cancer cell invasion, migration, and metastatic potential [33,35]. Mechanistic investigations suggest DDR2 promotes epithelial–mesenchymal transition, enhances matrix metalloproteinase secretion, and activates pro-survival signaling in colorectal cancer cells [18,33]. Despite these observations, several fundamental questions remain unresolved. First, whether DDR1 and DDR2 play distinct or redundant roles during colorectal cancer progression has not been systematically evaluated. Second, whether pathway activation occurs through classical receptor upregulation or through alternative mechanisms such as enhanced signaling efficiency remains unclear. Third, the identity of the most critical DDR target genes driving matrix remodeling in colorectal cancer has not been established, and it is unknown whether these relationships can be validated through orthogonal analytical approaches.

Type XI collagen is encoded by COL11A1, COL11A2, and COL2A1 genes. It represents a minor fibrillar collagen that normally comprises less than 3% of total collagen in most tissues, but which plays disproportionate roles in regulating collagen fibril assembly, diameter, and mechanical properties [36,37]. COL11A1 expression is restricted primarily to cartilage and developing tissues under physiological conditions, with minimal expression in adult epithelial organs [38,39]. However, recent studies have identified COL11A1 as among the most consistently and dramatically upregulated genes across diverse cancer types, including ovarian, pancreatic, head and neck, and colorectal cancers [40,41,42]. Elevated COL11A1 expression correlates with poor prognosis, chemotherapy resistance, and metastatic potential across multiple malignancies. Mechanistic studies suggest that COL11A1 promotes cancer progression through multiple pathways, including regulation of TGF-β signaling, modulation of integrin-mediated cell adhesion, and creation of a pro-metastatic tumor microenvironment [40,43]. Whether COL11A1 represents a direct transcriptional target of DDR2 signaling and whether DDR2-COL11A1 coupling drives colorectal cancer progression are issues that have not yet been investigated.

Traditional approaches to pathway analysis in cancer research rely primarily on the use of differential expression profiling to identify upregulated or downregulated genes or on pathway enrichment analysis to detect coordinated changes in functionally related gene sets. While powerful, these approaches capture only quantitative changes in expression levels and do not assess qualitative changes in transcriptional coordination between pathway components. Coexpression analysis quantifies pairwise correlations between genes, providing complementary information about regulatory coupling strength that can reveal how tightly coordinated gene expression programs become during disease progression [44,45]. Stage-stratified coexpression analysis, in which correlation patterns are compared across disease stages, enables detection of dynamic network reorganization that would be invisible to differential expression analysis alone [46,47]. However, correlation-based findings require validation through orthogonal approaches to distinguish genuine functional relationships from statistical artifacts.

The advent of interpretable machine learning methods offers powerful complementary approaches for validating transcriptional relationships identified through correlation analysis, particularly when deep neural networks are coupled with model explanation frameworks such as SHAP (SHapley Additive exPlanations) [48,49]. Deep learning models can learn complex nonlinear decision boundaries from high-dimensional gene expression data to classify disease states with high accuracy, while interpretability methods can identify which individual genes and gene interactions contribute most strongly to classification decisions [50,51]. When multiple independent analytical frameworks converge on the same genes or gene relationships, in this case correlation analysis, differential expression, and interpretable machine learning, confidence that these represent biologically meaningful signals rather than methodological artifacts is substantially increased.

In this study, we performed integrated computational analysis of 680 colorectal tissue samples spanning normal mucosa, adenomatous polyps, and invasive carcinoma in order to characterize DDR-mediated transcriptional network dynamics during tumour progression. Through stage-stratified correlation analysis of a fourteen-gene pathway encompassing DDR receptors, collagen genes, and matrix metalloproteinases, we quantified how transcriptional coupling evolves across the adenoma-to-carcinoma sequence. Deep neural network classification coupled with SHAP-based interpretability analysis was employed as an orthogonal computational framework to identify which gene relationships most strongly discriminate disease stages. The specific aims of this study were: (1) to determine whether DDR1 and DDR2 play distinct or overlapping roles in the transcriptional coordination of matrix remodeling genes during colorectal cancer progression; (2) to examine whether pathway activation is driven by receptor-level upregulation or by qualitative changes in transcriptional coupling efficiency; (3) to identify the most critical DDR2–target gene relationships for cancer-stage discrimination through convergent independent analytical methods; and (4) to characterize the temporal dynamics of pathway activation across the normal-to-adenoma and adenoma-to-carcinoma transition stages, with the aim of identifying potential windows for experimental intervention.

## 2. Results

### 2.1. Divergent Evolution of DDR1 and DDR2 Networks During Colorectal Tumorigenesis

To characterize DDR-mediated transcriptional coordination during colorectal cancer progression, we performed stage-stratified correlation analysis of a fourteen-gene pathway encompassing DDR receptors (DDR1, DDR2), collagen genes (COL1A1, COL1A2, COL3A1, COL5A1, COL5A2, COL11A1, FN1), and matrix metalloproteinases (MMP1, MMP2, MMP7, MMP9, MMP11) across 680 samples spanning normal mucosa (n=158), adenoma (n=170), and carcinoma (n=352) stages.

Global analysis of pairwise correlations revealed progressive integration of the pathway network during tumorigenesis (Appendix A.1). Normal tissue exhibited sparse correlation patterns with distinct modular architecture while carcinoma samples showed widespread positive associations across nearly all pathway genes, with the majority of gene pairs exceeding correlation coefficients of 0.5.

**Figure 1:**
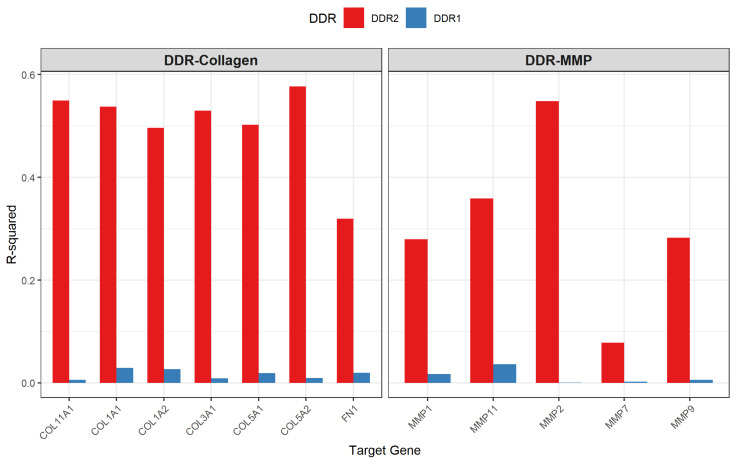
Stage-specific evolution of DDR1 and DDR2 correlation patterns with pathway targets. Comparison of coefficient of determination ($R^{2}$) values for DDR1 (blue) and DDR2 (red) correlations with collagen genes (**left panel**) and matrix metalloproteinases (**right panel**) in carcinoma samples (*n* = 352). DDR2 demonstrates strong correlations with all target genes ($R^{2}$ range: 0.08–0.58), while DDR1 shows uniformly weak correlations ($R^{2}<0.05$ for all genes). The divergence is particularly pronounced for COL5A2 (DDR2: $R^{2}=0.58$; DDR1: $R^{2}=0.01$), MMP2 (DDR2: $R^{2}=0.55$; DDR1: $R^{2}=0.04$), and COL11A1 (DDR2: $R^{2}=0.55$; DDR1: $R^{2}=0.01$), demonstrating DDR2-specific transcriptional coupling with matrix remodeling programs.

Focused examination of DDR-specific relationships revealed profound functional divergence between the two receptor family members (Figure 1). DDR1 correlations with downstream targets remained uniformly weak across all disease stages, with mean $R^{2}$ values actually declining from 0.031 in normal mucosa to 0.021 in adenoma and 0.017 in carcinoma, representing an overall reduction of 45%. In striking contrast, DDR2 demonstrated systematic strengthening of correlations, with mean $R^{2}$ increasing from 0.147 in normal tissue to 0.234 in adenoma and 0.380 in carcinoma, for a 2.59-fold overall enhancement. The DDR2-to-DDR1 correlation ratio expanded from 4.7-fold in normal tissue to 22.4-fold in carcinoma, demonstrating progressive functional specialization wherein DDR2 assumes the dominant role in coordinating matrix remodeling gene expression.

**Figure 2:**
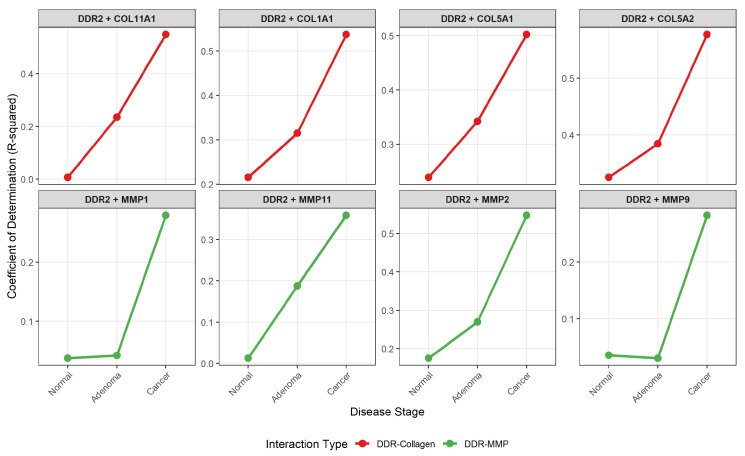
Progressive evolution of DDR2-driven coexpression. Temporal trajectories showing coefficient of determination ($R^{2}$) across normal, adenoma, and carcinoma stages for eight DDR2 pairs exhibiting strongest adenoma-to-carcinoma strengthening. DDR2-collagen interactions (red lines, top row) demonstrate progressive strengthening from varying baselines, with DDR2-COL5A2 showing the strongest correlation at all stages ($R^{2}$: 0.33 → 0.38 → 0.58) and DDR2-COL11A1 exhibiting near-complete de novo activation ($R^{2}$: 0.01 → 0.24 → 0.55). DDR2-MMP interactions (green lines, bottom row) predominantly show de novo activation patterns, with minimal baseline correlation followed by stepwise strengthening. All eight pairs exhibit steep slopes during the adenoma-to-carcinoma transition, indicating that critical pathway activation occurs during malignant transformation rather than early adenoma formation.

Among individual DDR2-to-target relationships, DDR2-COL11A1 exhibited the most dramatic coupling intensification, increasing from $R^{2}=0.007$ in normal tissue to $R^{2}=0.235$ in adenoma and $R^{2}=0.549$ in carcinoma (Figure 2). Additional DDR2 pairs showing substantial strengthening during adenoma-to-carcinoma transition included DDR2-MMP2 ($\Delta R^{2}=0.279$, 103% increase), DDR2-COL1A1 ($\Delta R^{2}=0.223$, 70% increase), and DDR2-MMP11 ($\Delta R^{2}=0.171$, 91% increase). Complete quantification of correlation changes for all 24 DDR-driven pairs is provided in Appendix A.2.

Detailed temporal trajectories for all strongly coexpressed DDR2 pairs are presented in Appendix A.3, revealing two distinct kinetic classes: Class I pairs (e.g., DDR2-COL5A2) showing progressive strengthening from detectable baselines, and Class II pairs (e.g., DDR2-COL11A1, DDR2-MMP11) exhibiting de novo activation from near-zero starting points.

### 2.2. Enhanced Transcriptional Coupling Despite Stable Receptor Expression

Differential expression analysis across disease stages revealed a critical dissociation between receptor abundance and pathway activity (Appendix A.4). Among 14 fourteen pathway genes, twelve showed significant upregulation in carcinoma versus normal tissue (Tukey HSD, p<0.05). COL11A1 demonstrated the most profound upregulation (1.99-fold, $p<10^{-15}$), followed by MMP7 (1.94-fold), MMP1 (1.80-fold), and MMP11 (1.49-fold). Collagen genes showed moderate upregulation, with COL5A2 increasing 1.18-fold ($p<10^{-15}$).

**Figure 3:**
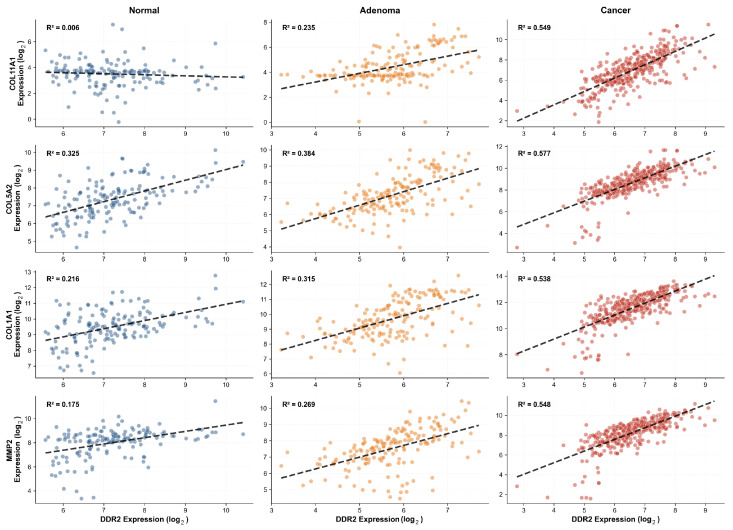
Progressive strengthening of DDR2-driven coexpression networks across colorectal tumorigenesis. Scatter plots with regression lines show DDR2 expression correlations with four key extracellular matrix targets across disease stages: normal mucosa (blue), adenoma (orange), and carcinoma (red). Each panel displays coefficient of determination ($R^{2}$) quantifying correlation strength. Top row: DDR2-COL11A1 interaction shows dramatic strengthening from essentially no correlation in normal tissue ($R^{2}=0.006$) through moderate adenoma correlation ($R^{2}=0.235$) to strong cancer coexpression ($R^{2}=0.549$). Second row: DDR2-COL5A2 follows similar trajectory with most robust cancer correlation ($R^{2}=0.577$). Third and fourth rows: DDR2-COL1A1 and DDR2-MMP2 pairs demonstrate consistent pattern of progressive correlation tightening. The systematic strengthening across multiple targets indicates coordinated pathway activation through enhanced transcriptional coupling rather than individual gene dysregulation, with most dramatic intensification occurring during adenoma-to-carcinoma transition.

Remarkably, DDR2 expression exhibited a V-shaped pattern, declining 20% from normal mucosa (mean expression 7.21) to adenoma (5.74), then partially recovering in carcinoma (6.57), yielding net 0.91-fold change (slight downregulation). This occurred while DDR2-mediated coexpression networks strengthened 2.59-fold, as demonstrated through comprehensive scatter plot analysis (Figure 3). The progressive tightening of DDR2 correlations with multiple extracellular matrix components illustrates that pathway activation proceeds through enhanced regulatory coupling rather than receptor upregulation. Across all four major DDR2 interactions examined, correlation strengthening followed a consistent pattern: minimal coordination in normal tissue ($R^{2}=0.006$–0.325), emerging relationships in adenoma ($R^{2}=0.235$–0.384), and robust coexpression in carcinoma ($R^{2}=0.538$–0.577).

Similarly, MMP2 showed minimal transcriptional upregulation (1.03-fold, p=0.171) despite achieving strong coexpression with DDR2 in carcinoma ($R^{2}=0.548$). DDR1 showed only modest upregulation (1.04-fold) with no strengthening of target correlations. Category-wise expression analysis (Appendix A.5) revealed that matrix metalloproteinases showed the strongest average upregulation (mean 1.60-fold), while DDR receptors remained essentially stable.

**Figure 4:**
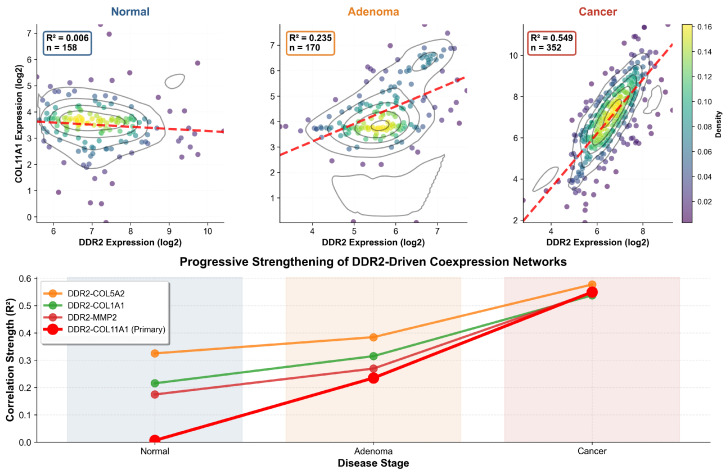
Density analysis and trajectory quantification of DDR2-COL11A1 coexpression strengthening. Upper panels: Contour density plots with scatter overlay visualize expression distribution and correlation patterns for DDR2-COL11A1 across normal mucosa, adenoma, and carcinoma. Points are colored by local density (viridis colormap), black contour lines delineate density levels, and red dashed lines show regression fit. Normal tissue exhibits diffuse, uncorrelated distribution with flat density landscape ($R^{2}=0.006$). Adenoma shows nascent density clustering and emerging correlation ($R^{2}=0.235$). Carcinoma demonstrates tight elliptical density concentration along steep regression trajectory ($R^{2}=0.549$), indicating highly coordinated coexpression. Lower panel: Trajectory plot quantifies correlation strengthening across stages for four DDR2 interactions. DDR2-COL11A1 (red line, primary focus) shows most dramatic strengthening trajectory with 91.5-fold $R^{2}$ increase from normal to cancer. DDR2-COL5A2 (orange) exhibits strongest baseline correlation in normal tissue but similar terminal cancer correlation. DDR2-COL1A1 (green) and DDR2-MMP2 (blue) follow parallel strengthening trajectories. Stage-colored background regions indicate disease progression. The combined density-trajectory analysis demonstrates that coexpression network activation proceeds through progressive regulatory coordination rather than stochastic gene dysregulation.

To further elucidate the primary DDR2-COL11A1 interaction mechanism, we performed density-based coexpression analysis combining contour visualization with trajectory quantification (Figure 4). Gaussian kernel density estimation revealed fundamental reorganization of the DDR2-COL11A1 expression relationship across tumorigenesis. In normal mucosa, expression values showed diffuse, essentially uncorrelated distribution with density contours indicating random covariation ($R^{2}=0.006$, n=158). The adenoma stage exhibited intermediate organization, with nascent density clustering along the regression trajectory and moderate correlation emergence ($R^{2}=0.235$, n=170). Carcinoma samples demonstrated tight density concentration along a steep regression line, with contour ellipses indicating highly coordinated expression ($R^{2}=0.549$, n=352). The progressive density consolidation quantified through the trajectory plot demonstrates that DDR2-COL11A1 coexpression strengthens 91.5-fold from normal to cancer ($R^{2}$ increase from 0.006 to 0.549), establishing this interaction as the dominant regulatory axis in the pathway.

The temporal dynamics reveal biphasic pathway activation: moderate strengthening during normal-to-adenoma transition (1.59-fold mean DDR2 correlation increase), followed by dramatic intensification during adenoma-to-carcinoma transition (1.62-fold additional increase). Comparison across all four DDR2 interactions reveals divergent strengthening kinetics, with DDR2-COL5A2 showing earlier activation (baseline $R^{2}=0.325$ in normal tissue) but similar terminal correlation in cancer ($R^{2}=0.577$) and DDR2-COL11A1 exhibiting later but more dramatic strengthening from near-zero baseline. Extended expression analysis of DDR2 and all top coexpressed targets (Appendix A.6) confirmed these divergent patterns, with COL11A1 showing dramatic progressive upregulation (mean expression: 3.61 → 4.84 → 7.18) and MMP11 displaying strong cancer-specific activation (5.28 → 5.83 → 7.85), while DDR2 remained stable or decreased. Hierarchical clustering of fold-changes across all transitions (Appendix A.7) identified a highly upregulated cluster comprising MMP7, MMP1, COL11A1, and MMP11, distinguishing these genes from more modestly regulated pathway components including DDR2 itself.

### 2.3. Deep Learning Validation Identifies DDR2-COL11A1 as Critical Interaction

To validate the functional significance of observed coexpression patterns through an independent analytical framework, we trained a deep neural network on the fourteen-gene pathway signature to classify disease stages. The model achieved 93.14% accuracy on held-out test data (n=102), with per-class performance of 88.0% recall for adenoma, 96.2% for carcinoma, and 91.7% for normal mucosa. Training dynamics demonstrated effective learning with convergence by epoch 30 and absence of overfitting (Appendix A.8). Confusion matrix analysis (Appendix A.9) revealed that no normal samples were misclassified as adenoma and vice versa, indicating a clear transcriptional boundary between non-neoplastic and neoplastic states. ROC analysis demonstrated outstanding discrimination, with AUC values of 0.981 (adenoma), 0.974 (carcinoma), and 0.970 (normal) (Appendix A.10).

**Figure 5:**
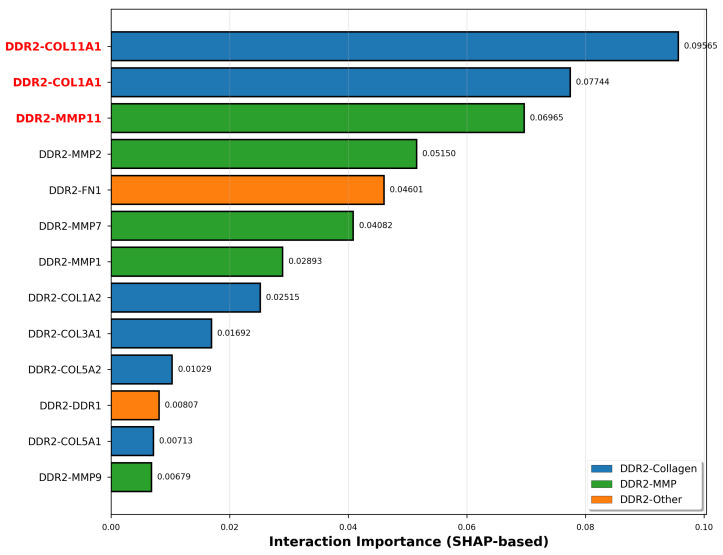
SHAP-based DDR2 interaction importance validates coexpression findings. Gene-gene interaction importance scores for all DDR2-target pairs in cancer classification, color-coded by interaction type: DDR2-collagen (blue), DDR2-MMP (green), DDR2-other (orange). The top three interactions—DDR2-COL11A1 (0.0956, highlighted in red text), DDR2-COL1A1 (0.0774), and DDR2-MMP11 (0.0697)—precisely match the gene pairs showing strongest coexpression strengthening in correlation analysis, providing independent validation through orthogonal methodology. DDR2-COL11A1 exhibits 23.5% higher interaction importance than DDR2-COL1A1, consistent with COL11A1 being the primary DDR2 target. The dominance of collagen and MMP interactions over DDR2-DDR1 (0.0081) demonstrates that DDR2 functions as a regulatory hub coordinating downstream matrix remodeling.

SHAP analysis identified which genes and gene interactions drive classification decisions (Figure 5). Individual gene importance ranked COL11A1 highest (SHAP = 0.506), followed by COL1A1 (0.410), MMP11 (0.367), MMP2 (0.269), and FN1 (0.259). DDR2 ranked seventh (0.182), while DDR1 ranked twelfth (0.048), confirming that receptor expression levels alone have modest discriminative power compared to downstream matrix remodeling genes. Complete feature importance rankings for all fourteen genes are provided in Appendix A.11.

Critically, DDR2 gene–gene interaction importance analysis revealed DDR2-COL11A1 as the top-ranked interaction (importance = 0.0956), 23.5% higher than the second-ranked DDR2-COL1A1 interaction (0.0774), with DDR2-MMP11 ranking third (0.0697). Complete interaction importance scores for all thirteen DDR2–target pairs are detailed in Appendix A.13. SHAP summary visualization (Appendix A.12) revealed that high expression of collagen and MMP genes generally corresponds to positive SHAP values driving cancer classification, while low expression shows negative contributions.

The convergence of three independent analytical approaches; correlation analysis showing DDR2-COL11A1 with strongest coupling intensification ($\Delta R^{2}=0.542$ from normal to cancer), differential expression revealing COL11A1 as most upregulated gene (1.99-fold), and deep learning identifying DDR2-COL11A1 as top interaction (SHAP = 0.0956) provides convergent multi-method computational evidence that this interaction represents a candidate transcriptional correlate of colorectal cancer progression rather than a statistical artifact.

## 3. Discussion

This study establishes enhanced transcriptional coupling efficiency rather than receptor upregulation as the primary mechanism driving DDR2-mediated matrix remodeling during colorectal cancer progression. Through convergent evidence from correlation analysis, differential expression profiling, and interpretable deep learning, we demonstrate that DDR2 coordinates an extracellular matrix remodeling program characterized by progressive strengthening of regulatory relationships with collagen and matrix metalloproteinase targets, while DDR1 does not. The identification of DDR2-COL11A1 as the critical gene interaction is validated independently by three orthogonal computational approaches, identifying a candidate relationship for experimental investigation that is inconsistent with simple receptor-centric models of oncogenic pathway activation.

### 3.1. Coupling Efficiency as a Candidate Mechanism: Transcriptional Evidence

The paradoxical observation that DDR2-mediated coexpression networks strengthen 2.59-fold while DDR2 expression remains stable or decreases fundamentally challenges the receptor abundance model that dominates cancer biology. Classical oncogenic receptor tyrosine kinases—EGFR in glioblastoma, HER2 in breast cancer, MET in gastric cancer—drive pathway activation through amplification or overexpression, creating direct proportionality between receptor levels and signaling output. DDR2 in colorectal cancer operates differently: pathway activation occurs through qualitative changes in signaling architecture rather than quantitative changes in receptor abundance.

This coupling efficiency model posits that the same DDR2 expression level exerts dramatically stronger regulatory control in cancer versus normal cells. Multiple mechanisms likely converge to optimize signal transduction. Post-translational modifications such as phosphorylation, palmitoylation, and glycosylation could enhance DDR2 specific activity or membrane localization without changing transcript levels. Downstream pathway reorganization may amplify weak DDR2 signals through increased expression of immediate effectors (Src family kinases, PI3K, MAPK components) or recruitment of transcriptional cofactors at target gene promoters. Epigenetic remodeling of target genes (loss of H3K27me3/H3K9me3 repressive marks, gain of H3K4me3/H3K27ac activating marks) could prime chromatin for rapid transcriptional response to DDR2 signaling. Loss of negative feedback mechanisms such as downregulation of phosphatases, ubiquitin ligases, transcriptional repressors may sustain pathway activity. The collagen-rich tumor microenvironment itself provides sustained ligand stimulation, driving maximal receptor occupancy at physiological expression levels.

If validated experimentally, the coupling efficiency model would have potential therapeutic implications. The hypothesis that cancer cells may depend on optimized DDR2 transcriptional output despite normal receptor abundance could suggest that DDR2 inhibition might achieve selectivity through differential pathway dependency. This remains speculative based on transcriptional data alone; functional studies in patient-derived organoids or xenograft models are required to test whether enhanced transcriptional coupling corresponds to increased vulnerability to DDR2 disruption. These computational observations may inform patient stratification hypotheses; however, clinical translation requires validation of the coupling efficiency concept at the protein and functional level.

### 3.2. Functional Specialization: DDR2 Dominance and DDR1 Suppression

The 22.4-fold divergence in pathway coupling between DDR2 and DDR1 in carcinoma reveals unexpected functional specialization within the DDR family. DDR1 correlations actively declined 45% during progression, with several relationships showing paradoxical weakening during malignant transformation (DDR1-MMP1: $R^{2}$ declining from 0.099 to 0.017). This suggests active suppression rather than passive nonparticipation, potentially mediated by microRNA-dependent mRNA destabilization, epigenetic silencing of DDR1 target genes, or competition for limiting transcriptional cofactors.

The molecular basis for DDR2 dominance likely reflects convergent advantages. DDR2’s higher affinity for fibrillar collagens (types I and III) matches the collagen-rich tumor microenvironment. Unique DDR2 phosphorylation sites recruit specific effectors (Src family kinases, ShcA) that are particularly effective at driving matrix remodeling programs. DDR2 expression in cancer-associated fibroblasts may mediate paracrine signaling coordinating stromal–epithelial remodeling, while epithelial DDR1 serves homeostatic functions irrelevant to malignant transformation.

At the transcriptional level, this functional specialization suggests that DDR2-selective inhibitors merit investigation over pan-DDR approaches, as DDR1 transcriptional activity declines during progression. Whether this transcriptional divergence reflects functionally distinct protein-level activities requires experimental validation. These observations generate testable hypotheses for future in vitro and in vivo studies comparing selective DDR2 inhibition versus pan-DDR inhibition in colorectal cancer models.

### 3.3. DDR2-COL11A1: A Critical Therapeutic Axis

The convergence of three independent analytical approaches on DDR2-COL11A1 provides exceptional validation of this relationship’s functional importance. Correlation analysis revealed the most dramatic coupling intensification (78-fold increase from normal to cancer, final $R^{2}=0.549$). Differential expression identified COL11A1 as the most upregulated pathway gene (1.99-fold, near-doubling). Deep learning ranked DDR2-COL11A1 as the top interaction for cancer classification (SHAP = 0.0956, 23.5% higher than second-ranked). This multi-method convergence reduces the likelihood of method-specific artifacts and is consistent with, but does not prove, biological dependency.

COL11A1’s functional importance extends beyond structural roles. As a minor fibrillar collagen (<3% of total collagen) normally restricted to cartilage and developing tissues, its dramatic ectopic upregulation in colorectal cancer represents recruitment of a silenced developmental program. Type XI collagen regulates fibril diameter and organization, potentially creating matrix architectures favoring tumor cell migration. Cryptic bioactive domains exposed by proteolytic processing may generate matricryptic peptides with pro-migratory or pro-angiogenic activities. COL11A1-mediated integrin signaling may function independently of structural matrix assembly.

COL11A1’s restricted normal expression combined with robust transcriptional upregulation in carcinoma suggests a potential differential expression window that warrants experimental investigation. The transcriptional coupling between DDR2 and COL11A1 generates the hypothesis that DDR2 inhibition may secondarily reduce COL11A1 expression; this remains to be tested functionally. The consistent association between high COL11A1 expression and adverse clinical outcomes reported across cancer types including ovarian, pancreatic, and head and neck cancers supports prioritization of this gene for experimental validation, but transcriptional upregulation alone is insufficient to designate COL11A1 a therapeutic target without functional evidence.

### 3.4. Temporal Dynamics and Intervention Opportunities

The biphasic activation pattern consisting of moderate strengthening during adenoma formation (1.59-fold) followed by dramatic intensification during malignant transformation (1.62-fold additional) defines intervention opportunities. Early activation during adenoma formation reflects microenvironmental changes: cancer-associated fibroblast expansion producing collagen-rich ligand environments, inflammatory cytokine modulation of DDR2 signaling, and epigenetic priming of target genes. This establishes pathway foundation, but remains insufficient for full activation.

The dramatic intensification during adenoma-to-carcinoma transition coincides with acquisition of invasive capacity and likely reflects genetic alterations common in colorectal cancer. KRAS mutations (40% of cases) constitutively activate MAPK signaling, amplifying DDR2-initiated transcriptional responses. TP53 loss removes transcriptional repression and cell cycle constraints. Extensive epigenetic reprogramming affects DDR2 target gene promoters. Hypoxia-driven HIF activation synergizes with DDR2 signaling. Selection pressures favor cells with optimized matrix remodeling capacity.

This temporal progression suggests stage-specific intervention strategies. Early intervention during the adenoma stage could prevent or delay malignant transformation, representing chemoprevention in high-risk individuals with large or advanced adenomas. Late intervention in established carcinoma could block invasion and metastasis, which are the primary causes of mortality, potentially converting aggressive to indolent disease even without complete eradication. Biomarker-guided patient selection using DDR2 pathway activity signatures (high DDR2-target coexpression scores, elevated COL11A1 expression, or stromal collagen features) could identify those candidates most likely to benefit.

### 3.5. Methodological Advances and Future Directions

The convergence of correlation analysis, differential expression, and interpretable deep learning on identical gene relationships demonstrates the power of multi-method validation. Each approach has distinct assumptions and biases: correlation analysis detects coordinated regulation but lacks disease relevance; differential expression identifies transcriptional changes but misses regulatory coupling; deep learning captures complex patterns but lacks inherent interpretability. The fact that all three independently identify DDR2-COL11A1 as the critical relationship eliminates method-specific artifacts and provides exceptional confidence in biological validity.

This integrated computational framework could be applied broadly. Pan-cancer analysis across tumor types could determine whether coupling efficiency represents a general mechanism or colorectal-specific phenomenon. Integration of single-cell RNA sequencing would resolve cell type-specific contributions, distinguishing epithelial from stromal DDR2 functions. Spatial transcriptomics could map pathway activation heterogeneity within tumors, potentially revealing spatial organization of matrix remodeling programs. Longitudinal studies tracking pathway dynamics during tumor evolution could identify critical transition points optimal for intervention.

Critical experimental validation is required before the hypotheses generated here can be considered established. The following specific experiments are proposed as the immediate next steps: (1) DDR2 knockdown by siRNA or CRISPR-Cas9 in HCT116, SW480, and HT29 colorectal cancer cell lines, with quantification of COL11A1, MMP11, and other top coexpressed genes by qRT-PCR and Western blot, in order to test whether DDR2 depletion disrupts the transcriptional coordination observed computationally; (2) COL11A1 immunohistochemistry in an independent clinical cohort with matched survival data to evaluate the prognostic potential of COL11A1 protein expression suggested by the transcriptional upregulation data; (3) DDR2 kinase inhibitor treatment (dasatinib, imatinib, or selective DDR2 inhibitors) in patient-derived colorectal cancer organoids in order to measure COL11A1 and matrix remodeling gene expression as primary endpoints; (4) co-immunoprecipitation or proximity ligation assay studies to determine whether DDR2 and COL11A1 physically interact in colorectal cancer tissue, addressing the absence of PPI evidence in STRING; and (5) single-cell RNA sequencing of colorectal cancer patient samples to resolve whether the DDR2-COL11A1 coexpression relationship is cell-intrinsic or reflects cell-type compositional changes. These experiments would convert the computational observations reported here into mechanistically validated findings.

### 3.6. Computational Hypotheses for Experimental Investigation

The preceding computational analyses generate several hypotheses that merit systematic experimental testing before any clinical translation can be considered. We emphasize that the following represents speculative inference from transcriptional data only; none of these directions constitute therapeutic recommendations or clinical guidance.

At the level of receptor biology, the coupling efficiency model predicts that DDR2 kinase inhibition should produce disproportionate suppression of downstream matrix gene expression relative to receptor expression levels. This prediction could be tested in DDR2 inhibitor dose-response experiments in colorectal cancer cell lines and patient-derived organoids, measuring COL11A1 and other matrix gene expression as endpoints. Several DDR2 kinase inhibitors including dasatinib and imatinib have reported preclinical activity; however, clinical efficacy data in colorectal cancer are limited, and translational relevance of the coupling efficiency model to drug response has not been established.

At the biomarker level, the DDR2-COL11A1 coexpression relationship identified computationally could be evaluated as a candidate prognostic biomarker in independent clinical cohorts with survival data, using immunohistochemistry for protein-level validation. Gene expression signatures capturing DDR2 pathway transcriptional activity represent a hypothesis for biomarker development, but require prospective clinical validation before any application to patient stratification.

The temporal staging of transcriptional network activation suggests examining DDR2 pathway activity in adenoma versus carcinoma in experimental progression models. Whether the transcriptional changes observed reflect causal drivers or passenger correlates of progression cannot be determined from observational transcriptomics; causal inference requires genetic manipulation experiments.

### 3.7. Limitations

This study has several important limitations that must be acknowledged. First and most fundamentally, the analysis is entirely computational. All conclusions are derived from transcriptomic data and represent statistical associations and patterns in gene expression. The study does not include protein-level validation, functional assays, cell line experiments, animal models, or patient samples beyond the reanalyzed public microarray datasets. Transcriptional coexpression is a distinct biological phenomenon from direct protein–protein interaction; the progressive strengthening of DDR2-COL11A1 coexpression across disease stages is a transcriptional observation that does not establish physical interaction, causal regulatory control, or functional dependency between these proteins.

Second, the fourteen-gene DDR-collagen-MMP pathway was defined through literature review prior to analysis, introducing potential selection bias. Genes were included based on known biology rather than being discovered de novo through unbiased analysis. While this approach focused the analysis on biologically motivated relationships, it cannot exclude the possibility that other gene sets would show equally strong or stronger coexpression patterns. The pathway definition circularity—selecting genes based on known interactions and then analyzing their coexpression—limits the novelty of findings for well-established relationships within the set, although the stage-specific dynamics and the DDR1 versus DDR2 divergence were not predetermined.

Third, while batch correction using ComBat was applied to the five integrated GEO datasets, residual technical variation from dataset-specific factors such as patient population, sample collection protocols, and microarray hybridization conditions cannot be fully excluded. The consistency of findings across subsets of the data provides some reassurance, but independent validation in a prospectively collected cohort would strengthen confidence.

Fourth, while the deep learning classification model achieved high accuracy (93.14%) on held-out test data, it was trained and evaluated on data from the same five GEO datasets used for coexpression analysis. External validation on fully independent datasets would be required to assess generalizability. SHAP interaction importance scores reflect the model’s learned decision patterns, and should be interpreted as data-driven hypotheses rather than confirmed biological mechanisms.

Fifth, as noted in the Methods section, the study analyzed bulk microarray data from heterogeneous tissue samples containing mixtures of epithelial, stromal, immune, and vascular cells. DDR2 is expressed in cancer-associated fibroblasts as well as tumor epithelial cells; thus, the observed coexpression patterns may reflect cell type compositional changes across disease stages rather than or in addition to cell-intrinsic transcriptional regulation. Single-cell resolution would be required in order to resolve cell-type-specific contributions.

Finally, STRING v12.0 analysis of the fourteen pathway genes confirmed that DDR2-COL11A1 has zero experimental protein–protein interaction evidence, consistent with this being a transcriptionally defined rather than biochemically characterized relationship. The absence of PPI evidence does not invalidate the coexpression finding, but underscores that this study describes a transcriptional pattern requiring protein-level and functional characterization.

Beyond the technical limitations described above, several biological and pathophysiological processes unrelated to colorectal tumorigenesis may influence DDR2-COL11A1 pathway activation and the coexpression relationships reported here. DDR2 is activated wherever fibrillar collagen is abundant; COL11A1 is induced in fibrotic, inflammatory, and wound healing contexts independently of cancer. Comorbidities prevalent in colorectal cancer patient populations, including inflammatory bowel disease, hepatic steatosis, obesity, and age-related fibrotic changes, were not documented in the GEO dataset records used here, and as such could not be controlled for in the analysis. The extent to which conditions like these contribute to the observed transcriptional patterns, particularly in normal mucosa and adenoma samples, is unknown. This further underscores the necessity of prospective experimental validation in well-characterized patient cohorts with complete clinical metadata before any causal interpretation of the DDR2-COL11A1 coexpression relationship can be made.

## 4. Materials and Methods

### 4.1. Data Acquisition and Preprocessing

Gene expression data for coexpression and differential expression analyses were obtained from the Gene Expression Omnibus public repository (GEO, https://www.ncbi.nlm.nih.gov/geo/, accessed on 3 January 2026). Five independent datasets profiled on the Affymetrix Human Genome U133 Plus 2.0 Array platform (GPL570) were selected in order to enable cross-platform integration without technical batch effects from differing microarray chemistries. The datasets comprised GSE20916, GSE41258, GSE4183, GSE77953, and GSE8671, collectively providing matched normal colonic mucosa, adenomatous polyp, and colorectal carcinoma samples from 680 unique patients across multiple institutions and geographic regions. GSE41258, the largest cohort, contributed 390 samples including 74 normal mucosa, 51 adenoma, and 265 carcinoma specimens, providing substantial representation of the adenoma-to-carcinoma sequence. GSE20916 provided 145 samples with balanced representation across all three disease stages (44 normal, 55 adenoma, 46 carcinoma), enabling robust stage-comparative analyses. GSE8671 contributed 64 samples comprising 32 normal mucosa and 32 matched adenoma samples from the same individuals, enabling paired analysis of early neoplastic transformation. GSE77953 provided 58 samples focusing on adenoma-carcinoma transition with 17 adenoma and 41 carcinoma specimens. GSE4183 contributed 23 samples including 8 normal and 15 adenoma specimens representing early neoplastic changes. After quality control filtering to remove samples with poor hybridization quality or incomplete clinical annotation, the final integrated dataset comprised 158 normal mucosa samples, 170 adenoma samples, and 352 carcinoma samples, providing substantial statistical power for detection of stage-specific transcriptional changes (Table 1).

The clinical characteristics of the patient cohorts contributing to each dataset are constrained by the metadata deposited by original investigators in GEO records. Patient age and sex were not consistently documented across all five datasets, and consequently are not available for the integrated cohort as a whole. Within the carcinoma group, disease stage varied across contributing datasets: GSE41258, the largest contributor, includes colorectal carcinoma samples spanning stages I through IV as well as a subset of liver metastasis specimens from surgically resected patients, while GSE20916 similarly encompasses stages I through IV. Adenoma samples across GSE4183, GSE8671, and GSE77953 represent tubular and tubulovillous polyps identified at routine colonoscopy. Normal mucosa samples were derived from histologically confirmed non-neoplastic colonic tissue, obtained either as matched normal–adenoma paired biopsies from the same individual (GSE8671) or as separate normal control specimens (remaining datasets).

Raw microarray data files in CEL format were downloaded and processed using the Robust Multi-array Average (RMA) normalization algorithm as implemented in the affy package (version 1.74.0) for R statistical software (version 4.2.0). The RMA procedure performs background correction using a convolution model that adjusts perfect match probe intensities for non-specific binding, uses quantile normalization to enforce identical intensity distributions across all arrays in order to remove systematic technical variation, and summarizes probe set intensities using median polish to generate gene-level expression values. Following RMA normalization, expression values were log2-transformed to achieve approximate normality and stabilize variance across the expression range, yielding values typically ranging from 3 to 15 on the log2 scale, corresponding to approximately 8-fold to 32,000-fold above background. Probe sets were annotated to gene symbols using the hgu133plus2.db annotation package (version 3.13.0), with probe sets mapping to multiple genes excluded from analysis to avoid ambiguity and multiple probe sets mapping to the same gene averaged to generate single representative expression values per gene.

Batch effect correction was performed using the ComBat algorithm from the sva package (version 3.44.0), which employs empirical Bayes methods to adjust for known batch effects while preserving biological variation. ComBat models the expression data as

(1) $$Y_{ijg}=\alpha_{g}+X\beta_{g}+\gamma_{ig}+\delta_{ig}\epsilon_{ijg},$$

where $Y_{ijg}$ is the expression of gene *g* for sample *j* in batch *i*, $\alpha_{g}$ is the overall gene expression, $X\beta_{g}$ represents biological covariates including disease stage (normal, adenoma, carcinoma), $\gamma_{ig}$ is the additive batch effect, $\delta_{ig}$ is the multiplicative batch effect, and $\epsilon_{ijg}$ is the error term. The algorithm estimates batch effects using empirical Bayes shrinkage to improve stability when batch sizes are small, then adjusts expression values to remove estimated batch effects while retaining biological signal associated with disease stage. We specified dataset of origin as the batch variable and disease stage as the biological covariate to preserve, verified batch correction efficacy through principal component analysis to confirm that the first two principal components separated samples by disease stage rather than dataset of origin post-correction, and validated that known biological signals including established colorectal cancer markers such as CEA, MYC, and CCND1 showed expected upregulation patterns after correction.

### 4.2. Gene Selection and Pathway Definition

The DDR–collagen–MMP pathway was defined based on comprehensive literature review of discoidin domain receptor biology, extracellular matrix remodeling mechanisms, and matrix metalloproteinase regulation in cancer. The pathway comprised 14 genes selected to represent three functional categories: DDR receptors including DDR1 and DDR2 which serve as collagen-activated receptor tyrosine kinases mediating cellular responses to matrix composition, collagen genes including COL1A1, COL1A2, COL3A1, COL5A1, COL5A2, COL11A1, and FN1 that represent major fibrillar collagens along with associated matrix proteins that serve as DDR ligands and structural matrix components, and matrix metalloproteinases including MMP1, MMP2, MMP7, MMP9, and MMP11 representing key enzymes mediating collagen degradation and matrix remodeling. This 14-gene pathway encompasses the complete regulatory circuit from receptor activation through target gene expression to functional matrix remodeling output, enabling comprehensive assessment of pathway coordination.

Gene expression values for all 14 pathway genes were extracted from the preprocessed and batch-corrected expression matrices for both coexpression and survival datasets. Quality control of gene-specific expression distributions confirmed that all genes showed adequate dynamic range with coefficient of variation exceeding 0.2 across samples, indicating sufficient biological variability for meaningful correlation analysis. Visual inspection of expression distributions confirmed approximate normality of log2-transformed values for most genes, with some genes including MMP7 and MMP1 showing bimodal distributions likely reflecting differential activation in tumor versus normal tissue subsets. No genes were excluded based on expression quality control, as all 14 pathway members showed robust detection above background across the majority of samples.

### 4.3. Coexpression Network Analysis

Pairwise correlation analysis was performed to quantify transcriptional coordination among all possible gene pairs within the 14-gene pathway. For each disease stage (normal mucosa, adenoma, carcinoma), Pearson correlation coefficients were calculated for all 142=91 unique gene pairs using the cor function in R. The Pearson correlation coefficient between genes *i* and *j* was computed as

(2) $$r_{ij}=\frac{\sum_{k=1}^{n}\left(x_{ik}-{\bar{x}}_{i}\right)\left(x_{jk}-{\bar{x}}_{j}\right)}{\sqrt{\sum_{k=1}^{n}{\left(x_{ik}-{\bar{x}}_{i}\right)}^{2}}\sqrt{\sum_{k=1}^{n}{\left(x_{jk}-{\bar{x}}_{j}\right)}^{2}}},$$

where $x_{ik}$ represents the log2 expression value of gene *i* in sample *k*, ${\bar{x}}_{i}$ is the mean expression of gene *i* across all *n* samples in the disease stage, and $r_{ij}$ ranges from −1 (perfect negative correlation) through 0 (no correlation) to +1 (perfect positive correlation). The coefficient of determination $R^{2}=r_{ij}^{2}$ was calculated to quantify the proportion of variance in one gene’s expression explained by the other gene’s expression, providing an interpretable metric of coupling strength ranging from 0 (no shared variance) to 1 (perfectly coupled expression). Statistical significance of correlations was assessed using the cor.test function which implements *t*-tests under the null hypothesis of zero correlation, with *p*-values adjusted for multiple testing using the Benjamini–Hochberg false discovery rate method applied separately within each disease stage to control the expected proportion of false positives among declared significant correlations at the 5% FDR threshold.

For DDR-specific analyses focusing on the two receptor family members, we extracted all correlation coefficients involving DDR1 or DDR2 with their 12 downstream target genes (excluding correlations between DDR1 and DDR2 themselves). This generated 24 DDR-driven correlations per disease stage, 12 for each of DDR1 and DDR2. Mean correlation strength for each receptor was computed by averaging $R^{2}$ values across its 12 target relationships, providing summary metrics of overall pathway coupling. Temporal trajectories of specific gene pairs were visualized by plotting $R^{2}$ values across the three disease stages (normal, adenoma, carcinoma) with connecting lines to illustrate progression patterns. Fold-change in correlation strength during specific transitions was calculated as the ratio of $R^{2}$ values between adjacent stages, for example adenoma-to-carcinoma strengthening as $R_{\mathrm{carcinoma}}^{2}/R_{\mathrm{adenoma}}^{2}$.

Hierarchical clustering of genes based on correlation patterns was performed using the hclust function, with average linkage and correlation distance metric defined as $d_{ij}=1-\left|r_{ij}\right|$ in order to treat positive and negative correlations symmetrically. This distance metric ensures that strongly correlated gene pairs (whether positively or negatively) cluster together, while weakly correlated pairs are separated. Dendrograms were generated to visualize hierarchical relationships and identify gene modules characterized by strong internal correlations and weak external correlations. Correlation matrices were visualized as heatmaps using the corrplot package, with color intensity representing correlation strength from blue (negative correlation) through white (zero correlation) to red (positive correlation) and hierarchical clustering dendrograms displayed on margins to show gene groupings.

### 4.4. Differential Expression Analysis

Statistical comparison of gene expression levels across disease stages was performed using one-way analysis of variance (ANOVA) to test the global null hypothesis that mean expression is equal across all three stages (normal, adenoma, carcinoma) for each gene independently. The ANOVA F-statistic for gene *g* was calculated as

(3) $$F_{g}=\frac{{MS}_{\text{between}}}{{MS}_{\text{within}}}=\frac{\sum_{s=1}^{3}n_{s}{\left({\bar{x}}_{gs}-{\bar{x}}_{g}\right)}^{2}/\left(3-1\right)}{\sum_{s=1}^{3}\sum_{i=1}^{n_{s}}{\left(x_{gsi}-{\bar{x}}_{gs}\right)}^{2}/\left(N-3\right)},$$

where *s* indexes the three disease stages, $n_{s}$ is the number of samples in stage *s*, $N=\sum_{s=1}^{3}n_{s}$ is the total sample size, $x_{gsi}$ is the expression of gene *g* in sample *i* of stage *s*, ${\bar{x}}_{gs}$ is the mean expression in stage *s*, and ${\bar{x}}_{g}$ is the overall mean across all stages. The F-statistic follows an F-distribution with degrees of freedom (3−1,N−3) under the null hypothesis, enabling calculation of *p*-values testing whether at least one stage differs in mean expression. Multiple testing correction was applied using the Benjamini–Hochberg FDR procedure across all 14 genes.

For genes showing significant overall ANOVA results (FDR-adjusted *p* < 0.05), pairwise post hoc comparisons between all stage combinations (normal versus adenoma, normal versus carcinoma, adenoma versus carcinoma) were performed using Tukey’s honest significant difference (HSD) test. Tukey’s HSD controls the family-wise error rate for multiple pairwise comparisons by adjusting the critical value based on the Studentized range distribution. For each gene, the test statistic for comparing stages $s_{1}$ and $s_{2}$ was

(4) $$q=\frac{{\bar{x}}_{gs_{1}}-{\bar{x}}_{gs_{2}}}{\sqrt{{MS}_{\text{within}}/2\cdot \left(1/n_{s_{1}}+1/n_{s_{2}}\right)}},$$

which follows the Studentized range distribution with parameters (3,N−3) under the null hypothesis of equal means. Tukey-adjusted *p*-values were calculated for each pairwise comparison, with significance declared at *p* < 0.05 after adjustment. Fold-changes between stages were calculated as $2^{\left({\bar{x}}_{gs_{2}}-{\bar{x}}_{gs_{1}}\right)}$ to convert log2 expression differences back to linear fold-change scale, with values greater than 1 indicating upregulation in the second stage and values less than 1 indicating downregulation.

### 4.5. Deep Neural Network Classification and Interpretability Analysis

To validate the functional significance of coexpression patterns and identify which genes and gene interactions drive disease stage discrimination, we employed deep neural network classification coupled with SHapley Additive exPlanations (SHAP) analysis for model interpretability. The 14-gene DDR2 pathway signature was used as input features to train a supervised classifier distinguishing normal mucosa, adenoma, and carcinoma samples, with model performance quantifying the discriminative information content of the pathway and SHAP values identifying the relative contribution of individual genes and gene pairs to classification decisions.

A feed-forward deep neural network was implemented using PyTorch (version 2.0.1) with Python 3.10. The network architecture comprised an input layer receiving 14 gene expression features, three hidden layers with 64, 32, and 16 nodes respectively, and an output layer with three nodes corresponding to the three disease stage classes. Each hidden layer employed rectified linear unit (ReLU) activation functions defined as f(x)=max(0,x) in order to introduce nonlinearity, enabling the network to learn complex decision boundaries while maintaining computational efficiency and avoiding vanishing gradient problems. Batch normalization was applied after each hidden layer to stabilize training by normalizing activations across mini-batches according to

(5) $$\hat{x}=\frac{x-\mu_{B}}{\sqrt{\sigma_{B}^{2}+\epsilon }},$$

where *x* is the input activation, $\mu_{B}$ and $\sigma_{B}^{2}$ are the mean and variance computed over the mini-batch, and $\epsilon =10^{-5}$ is a small constant for numerical stability. Batch normalization reduces internal covariate shift and enables higher learning rates. Dropout regularization was applied after each hidden layer with dropout probabilities of 0.3, 0.3, and 0.2 for the first, second, and third hidden layers, respectively, randomly setting activation values to zero during training with the specified probability to prevent over-reliance on specific neurons and improve generalization to unseen data.

The output layer produced non-normalized class scores (logits) that were converted to probability distributions using the softmax function

(6) $$p_{i}=\frac{e^{z_{i}}}{\sum_{j=1}^{3}e^{z_{j}}},$$

where $z_{i}$ is the logit for class *i* and $p_{i}$ represents the predicted probability of that class. The network was trained to minimize the cross-entropy loss, defined as

(7) $$L=-\frac{1}{N}\sum_{k=1}^{N}\sum_{i=1}^{3}y_{ki}\log(p_{ki}),$$

where *N* is the number of training samples, $y_{ki}$ is the binary indicator (0 or 1) of whether sample *k* belongs to class *i*, and $p_{ki}$ is the predicted probability of class *i* for sample *k*. This loss function penalizes confident incorrect predictions more severely than uncertain predictions, encouraging the network to output well-calibrated probabilities.

The 680 samples were randomly partitioned into training (70%, n=476), validation (15%, n=102), and test (15%, n=102) sets using stratified sampling to preserve disease stage proportions within each partition. Stratification was implemented using the train_test_split function from scikit-learn (version 1.3.0) with the stratify parameter set to the disease stage labels. This ensured that each partition contained approximately 25% adenoma, 52% carcinoma, and 23% normal samples, matching the overall dataset composition. The training set was used for gradient-based parameter optimization, the validation set was used for hyperparameter tuning and early stopping decisions, and the test set was held out completely until final evaluation to provide unbiased estimates of generalization performance.

Gene expression features were standardized to zero mean and unit variance using the StandardScaler from scikit-learn, with scaling parameters computed exclusively on the training set and then applied to validation and test sets to prevent data leakage. Standardization was performed according to

(8) $$x_{ij}^{\prime }=\frac{x_{ij}-\mu_{j}}{\sigma_{j}},$$

where $x_{ij}$ is the expression value of gene *j* in sample *i*, $\mu_{j}$ and $\sigma_{j}$ are the mean and standard deviation of gene *j* computed over training samples only, and $x_{ij}^{\prime }$ is the standardized value. This transformation ensures that all genes contribute to the model on comparable scales regardless of their baseline expression levels or dynamic ranges.

Network parameters (weights and biases) were optimized using the Adam optimizer, an adaptive learning rate method that computes individual learning rates for each parameter based on first and second moments of gradients. The Adam update rule for parameter θ at iteration *t* is

(9) $$\begin{array}{cc} m_{t} & =\beta_{1}m_{t-1}+\left(1-\beta_{1}\right)g_{t}\\ v_{t} & =\beta_{2}v_{t-1}+\left(1-\beta_{2}\right)g_{t}^{2}\\ {\hat{m}}_{t} & =\frac{m_{t}}{1-\beta_{1}^{t}}\\ {\hat{v}}_{t} & =\frac{v_{t}}{1-\beta_{2}^{t}}\\ \theta_{t} & =\theta_{t-1}-\alpha \frac{{\hat{m}}_{t}}{\sqrt{{\hat{v}}_{t}}+\epsilon }, \end{array}$$

where $g_{t}$ is the gradient at iteration *t*, $m_{t}$ and $v_{t}$ are exponential moving averages of gradients and squared gradients respectively with decay rates $\beta_{1}=0.9$ and $\beta_{2}=0.999$, α=0.001 is the initial learning rate, and $\epsilon =10^{-8}$ prevents division by zero. L2 weight regularization with coefficient $\lambda =10^{-5}$ was applied by adding ${\lambda ||\theta ||}^{2}$ to the loss function to penalize large parameter values and prevent overfitting.

Training proceeded in mini-batches of 32 samples, with the training set randomly shuffled before each epoch to decorrelate successive batches. Learning rate scheduling was implemented using ReduceLROnPlateau from PyTorch, which monitors validation set accuracy and reduces the learning rate by a factor of 0.5 when accuracy fails to improve for 15 consecutive epochs, enabling fine-tuning of parameters as the optimization approaches convergence. Early stopping was employed to prevent overfitting by monitoring validation accuracy and terminating training if accuracy failed to improve for 30 consecutive epochs. The model parameters yielding the highest validation accuracy were saved and used for final evaluation, ensuring that performance estimates reflect the best model configuration rather than potentially overfit final iteration parameters.

Classification performance on the test set was assessed using multiple complementary metrics. Overall accuracy was calculated as the proportion of correctly classified samples across all three disease stages. Per-class precision, recall (sensitivity), and F1-score were computed for each disease stage independently. For class *c*, precision is defined as

(10) $${\text{Precision}}_{c}=\frac{TP_{c}}{TP_{c}+FP_{c}},$$

where $TP_{c}$ is the number of true positives (samples correctly classified as class *c*) and $FP_{c}$ is the number of false positives (samples incorrectly classified as class *c*). Recall is defined as

(11) $${\text{Recall}}_{c}=\frac{TP_{c}}{TP_{c}+FN_{c}},$$

where $FN_{c}$ is the number of false negatives (samples of class *c* incorrectly classified as another class). The F1-score combines precision and recall as their harmonic mean:

(12) $$F1_{c}=2\cdot \frac{{\text{Precision}}_{c}\cdot {\text{Recall}}_{c}}{{\text{Precision}}_{c}+{\text{Recall}}_{c}}.$$

Receiver operating characteristic (ROC) curves were generated for each class using a one-versus-rest approach, plotting the true positive rate against the false positive rate at various probability thresholds. The area under the ROC curve (AUC) was calculated using the trapezoidal rule, providing a threshold-independent measure of discrimination ability with values ranging from 0.5 (random classification) to 1.0 (perfect classification). Confusion matrices were generated to visualize classification patterns, with rows representing true disease stages and columns representing predicted stages, normalized by row to show the proportion of each true class predicted as each possible class.

To identify which genes contribute most strongly to classification decisions and quantify gene–gene interaction importance, we employed SHAP (SHapley Additive exPlanations) analysis using the shap package (version 0.42.1) for Python. SHAP values provide a unified measure of feature importance based on cooperative game theory, attributing the prediction of each sample to the contribution of individual features by computing Shapley values from coalition game theory. For a model *f* and input features $x=(x_{1},\;\ldots ,\;x_{n})$, the SHAP value $\phi_{j}$ for feature *j* represents the average marginal contribution of feature *j* across all possible feature coalitions:

(13) $$\phi_{j}=\sum_{S\subseteq \{1,\ldots ,n\}\setminus \{j\}}\frac{|S|!(n-|S|-1)!}{n!}\left[f\left(S\cup \left\{j\right\}\right)-f\left(S\right)\right],$$

where *S* represents a coalition of features, f(S) is the model prediction using only features in *S*, and the sum iterates over all possible coalitions not containing feature *j*. This formulation ensures that SHAP values satisfy desirable properties including local accuracy (sum of SHAP values equals the model prediction), missingness (features not used in the model have zero SHAP values), and consistency (if a model changes to increase a feature’s contribution, its SHAP value does not decrease).

For neural network models, exact SHAP value computation is intractable, so we employed GradientExplainer, a gradient-based approximation method that uses integrated gradients along paths from reference samples to the samples being explained. A background dataset of 100 randomly selected training samples was used to establish baseline expectations for feature values, with SHAP values computed for 100 randomly selected test samples to enable statistical summarization while maintaining computational tractability. SHAP values were computed separately for each output class, yielding class-specific importance scores indicating whether elevated or decreased expression of each gene pushes predictions toward that class.

Feature importance for each gene was quantified as the mean absolute SHAP value across all test samples for the carcinoma class, reflecting the average magnitude of that gene’s contribution to cancer classification regardless of direction. Genes were ranked by this metric to identify the most discriminative features. SHAP summary plots were generated to visualize the distribution of SHAP values for each gene across samples, with point color indicating feature value (gene expression level) to reveal whether high or low expression drives classification in particular directions.

To quantify how DDR2 interactions with downstream targets contribute to classification, we computed interaction importance scores by analyzing coordinated SHAP value patterns. For each DDR2–target gene pair, we calculated the interaction importance as the mean absolute product of SHAP values:

(14) $$I_{DDR2,j}=\frac{1}{N}\sum_{k=1}^{N}\left|\phi_{DDR2}^{\left(k\right)}\cdot \phi_{j}^{\left(k\right)}\right|,$$

where $\phi_{DDR2}^{\left(k\right)}$ and $\phi_{j}^{\left(k\right)}$ are the respective SHAP values for DDR2 and gene *j* in sample *k*, while N=100 is the number of test samples analyzed. This metric captures the degree to which DDR2 and the target gene jointly influence classification decisions, with high values indicating that samples where both genes have large (positive or negative) SHAP values are common, suggesting functional interaction in determining disease stage. Interaction scores were computed for all 13 DDR2–target pairs and ranked to identify the most important relationships for cancer classification.

All analyses were performed using Python 3.10 with PyTorch 2.0.1, NumPy 1.24.3, pandas 2.0.2, scikit-learn 1.3.0, matplotlib 3.7.1, seaborn 0.12.2, and shap 0.42.1. Code was executed on systems equipped with NVIDIA CUDA-compatible GPUs to accelerate neural network training, with automatic mixed precision training enabled to reduce memory consumption and increase computational throughput while maintaining numerical stability.

### 4.6. STRING Protein–Protein Interaction Analysis

To characterize the protein–protein interaction (PPI) evidence supporting the 14-gene DDR-collagen-MMP pathway and address the fundamental distinction between transcriptional coexpression and physical protein interaction, we performed a systematic STRING v12.0 analysis restricted to the 14 pathway genes. Human STRING interaction data (*Homo sapiens*, taxon 9606) were obtained as locally downloaded flat files (9606.protein.links.detailed.v12.0.txtand 9606.protein.aliases.v12.0.txt) to ensure complete reproducibility independent of the web interface. All 14 pathway genes were successfully mapped to canonical STRING ENSP identifiers using HGNC-sourced aliases. Interactions were filtered to retain only those where both partners were among the 14 pathway genes, yielding a focused analysis of 91 possible unique pairwise interactions.

**Figure A13:**
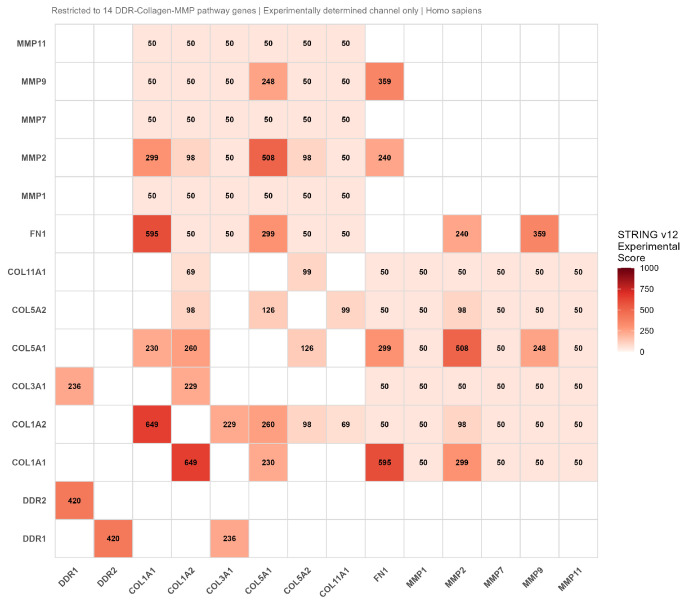
STRING v12.0 experimentally determined interaction scores among the fourteen DDR pathway genes. The heatmap displays experimentally determined interaction scores (biochemical evidence channel only, excluding text mining and coexpression channels) for all pairwise combinations of the fourteen DDR-collagen-MMP pathway genes in *Homo sapiens* (STRING v12.0). Scores range from 0 (no experimental evidence) to 1000 (maximum confidence); numerical values are shown only for pairs with experimental score >0. Of 91 possible unique pairwise interactions, 48 (52.7%) carry any experimental evidence, 4 (4.4%) reach the medium-confidence threshold (≥400), and 0 reach the high-confidence threshold (≥700) applied by the reviewer in the expanded 625-node network. Genes are ordered by functional category: DDR receptors (DDR1, DDR2), fibrillar collagens and FN1 (COL1A1–COL11A1), and matrix metalloproteinases (MMP1–MMP11). The DDR2–COL11A1 pair carries an experimental score of 0, confirming that this relationship has not been previously characterized at the protein–protein interaction level and represents a transcriptionally defined association identified in the present study.

**Figure A14:**
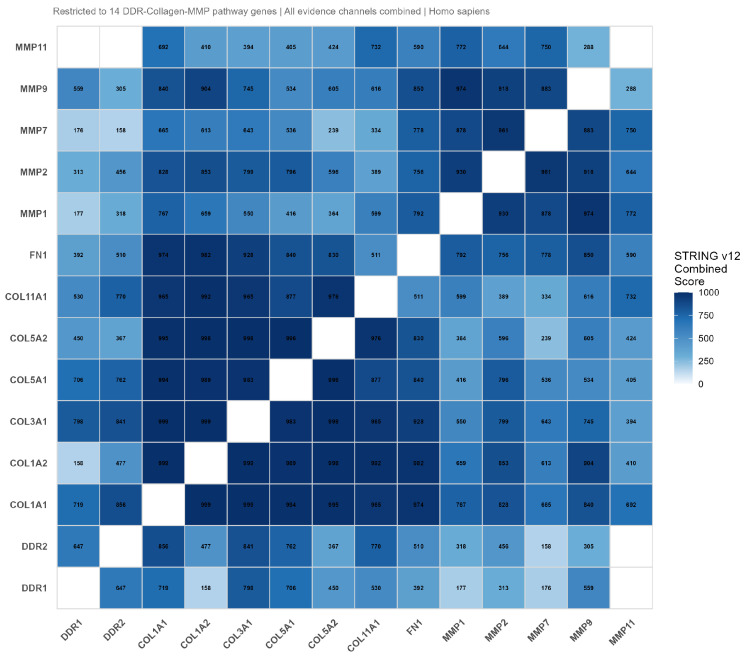
STRING v12.0 combined interaction scores (all evidence channels) Among the fourteen DDR pathway genes. The heatmap displays combined STRING confidence scores integrating all evidence channels (neighborhood, fusion, co-occurrence, coexpression, experimental, database, and text mining) for the same fourteen pathway genes. The combined scores are substantially higher than the experimentally determined scores alone, demonstrating that the DDR-collagen-MMP pathway is well-supported in the published literature. The DDR2-COL11A1 pair achieves a combined score of 770 (high-confidence range), driven primarily by text mining (754, reflecting systematic co-mention in the biomedical literature) and coexpression (105), with zero experimental and zero database evidence. This pattern confirms that while co-mentions of DDR2-COL11A1 are well-documented in the literature, physical protein–protein interaction has not been experimentally characterized, providing the scientific rationale for transcriptional coexpression analysis as a complementary and independent line of evidence.

In addition to the combined STRING score, evidence was assessed separately for the experimentally determined channel (biochemical experiments, co-immunoprecipitation, affinity capture) and the database/curated channel. Analysis at multiple experimental evidence thresholds (>0, ≥150, ≥400, ≥700, ≥900) was performed in order to characterize the landscape of physical interaction evidence within the pathway. Results are reported in Table A1 and visualized in Figure A13 and Figure A14.

## 5. Conclusions

This study provides convergent computational evidence consistent with DDR2-mediated transcriptional network reorganization as a candidate mechanism underlying extracellular matrix remodeling during colorectal cancer progression. Through integrated transcriptomic analysis of correlation dynamics, differential expression patterns, and deep learning-based classification with interpretability, we show that DDR2, but not DDR1, shows progressive strengthening of transcriptional coordination with collagen and matrix metalloproteinase genes across 680 patient samples spanning three disease stages. The observed dissociation between stable DDR2 receptor expression and progressively strengthening coexpression networks suggests, though it does not prove, that qualitative changes in transcriptional coupling efficiency may contribute to pathway dysregulation independently of receptor upregulation. This hypothesis warrants experimental investigation in functional models.

The temporal dynamics indicate biphasic transcriptional reorganization, with moderate coexpression strengthening during adenoma formation and dramatic intensification during malignant transformation, thereby defining potential windows for experimental intervention studies. Convergence of correlation analysis, differential expression, and deep learning interpretation on the DDR2-COL11A1 gene pair identifies this relationship as a priority for experimental investigation as a candidate biomarker of DDR2 transcriptional network activation. These findings challenge receptor-centric models of pathway dysregulation and suggest that future studies should examine post-translational regulatory mechanisms, epigenomic remodeling at target gene promoters, and the functional consequences of DDR2-COL11A1 transcriptional coupling in experimental colorectal cancer systems. Ultimately, this work provides a computational foundation for understanding how coordinated gene expression, rather than individual gene dysregulation, may characterize cancer-associated matrix remodeling, while also identifying specific testable hypotheses for wet-laboratory validation.

## Figures and Tables

**Table 1:** Gene expression datasets for coexpression and differential expression analysis.

Dataset	Platform	Normal	Adenoma	Carcinoma	Total	Characteristics
GSE20916	GPL570	44	55	46	145	Balanced stages
GSE41258	GPL570	74	51	265	390	Largest cohort
GSE4183	GPL570	8	15	0	23	Early neoplasia
GSE77953	GPL570	0	17	41	58	Adenoma-carcinoma
GSE8671	GPL570	32	32	0	64	Matched pairs
Total	–	158	170	352	680	–

**Figure A1:**
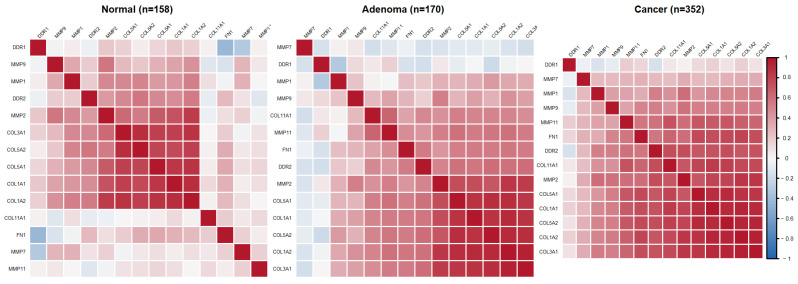
Stage-specific correlation matrices reveal progressive integration of the DDR-collagen-MMP network. Pearson correlation coefficients for all pairwise combinations of pathway genes are displayed as heatmaps for normal colonic mucosa (n=158), adenomatous polyps (n=170), and colorectal carcinoma (n=352). Hierarchical clustering based on correlation distance demonstrates distinct modular organization in normal tissue, with progressive network integration during malignant transformation. Color intensity represents correlation strength, with red indicating positive correlation, blue indicating negative correlation, and white representing absence of correlation. The dendrograms illustrate gene clustering patterns, revealing stage-specific coexpression modules. Quantitatively, normal tissue showed median correlations below 0.3, adenoma samples displayed intermediate values, and carcinoma samples showed the majority of gene pairs exceeding correlation coefficients of 0.5. Interactions linking DDR receptors to collagens and collagens to MMPs intensified significantly in cancer tissue.

**Figure A2:**
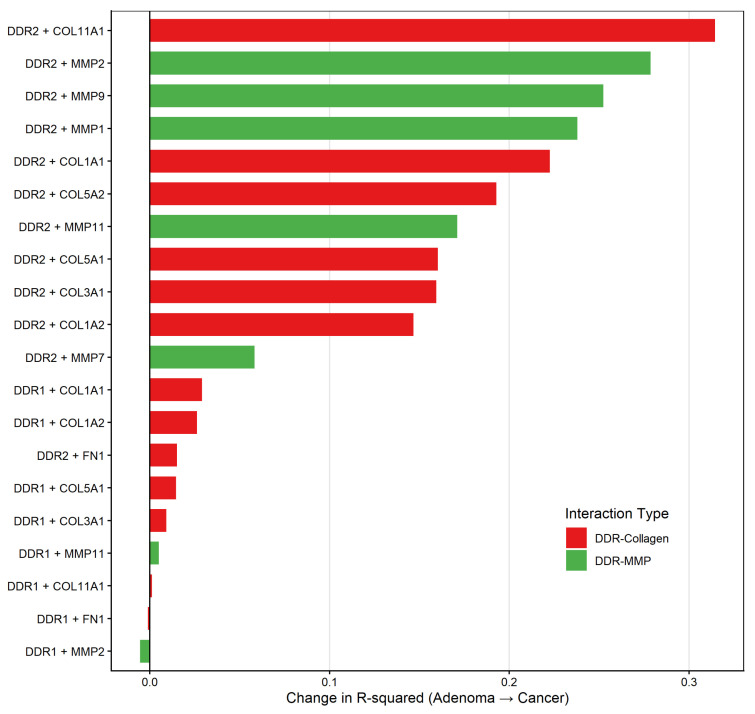
Magnitude of correlation change during the adenoma-to-carcinoma transition reveals DDR2 specificity. Change in coefficient of determination ($\Delta R^{2}$) is shown for all 24 DDR-driven pairs, ranked by strengthening magnitude. The top twelve positions are occupied exclusively by DDR2 pairs (DDR2-collagen in red, DDR2-MMP in green), with $\Delta R^{2}$ values ranging from 0.146 to 0.314. DDR2-COL11A1 shows the highest strengthening ($\Delta R^{2}=0.314$), followed by DDR2-MMP2 ($\Delta R^{2}=0.279$) and DDR2-MMP9 ($\Delta R^{2}=0.252$). DDR1 pairs (positions 13–24) show minimal strengthening or paradoxical weakening, with several exhibiting negative $\Delta R^{2}$ values. The clear separation between DDR2 and DDR1 pairs demonstrates that pathway intensification during malignant transformation is mediated specifically by DDR2 rather than representing a general DDR family phenomenon. The prominence of both DDR2-collagen and DDR2-MMP pairs in the top ranks indicates coordinated activation of both matrix synthesis and degradation arms of the remodeling program.

**Figure A3:**
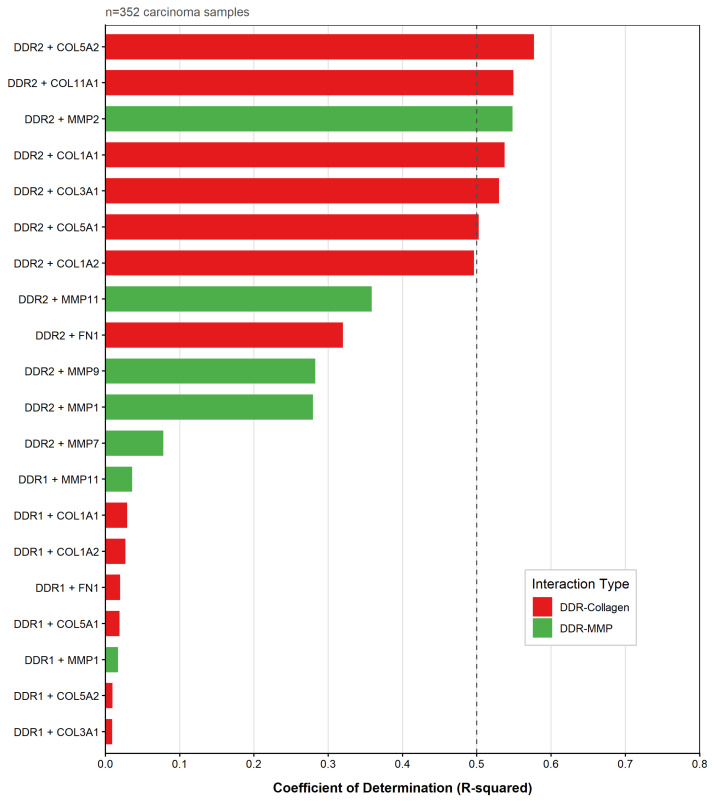
Ranking of top twenty DDR-driven gene pairs by correlation strength in carcinoma. The bar chart displays final $R^{2}$ values in carcinoma stage for the twenty most strongly correlated DDR-target relationships. DDR2 pairs (red/green) dominate the top positions, with DDR2-COL5A2 achieving the highest correlation ($R^{2}=0.577$), followed by DDR2-COL11A1 ($R^{2}=0.549$) and DDR2-MMP2 ($R^{2}=0.548$). Of the top fifteen positions, fourteen are occupied by DDR2 pairs, with only one DDR1 pair (DDR1-MMP11, $R^{2}=0.036$) appearing in the top twenty. This ranking demonstrates that in the malignant state, DDR2 has established strong regulatory relationships with multiple matrix remodeling targets, while DDR1 remains largely decoupled from the pathway.

**Figure A4:**
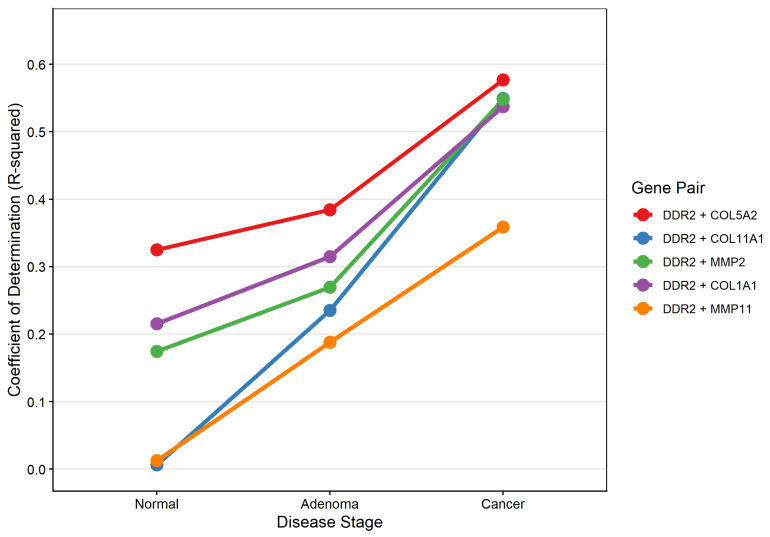
Detailed temporal trajectories of DDR2 pairs with strongest adenoma-to-carcinoma strengthening. Eight panels show $R^{2}$ values across normal, adenoma, and carcinoma stages for the most dynamically changing relationships. DDR-collagen pairs (red lines) generally demonstrate progressive strengthening from moderate baselines, while DDR-MMP pairs (green lines) often show minimal baseline correlation followed by stepwise activation. All eight pairs exhibit steep positive slopes between adenoma and carcinoma stages, contrasting with the flat or declining trajectories observed for DDR1 pairs. The coordinated upward trajectories indicate systematic network-wide intensification during tumor progression, with critical activation occurring during malignant transformation rather than during initial adenoma formation. Specific trajectories: DDR2-COL11A1: $R^{2}$ = 0.007 → 0.235 → 0.549 (78-fold overall increase). DDR2-COL1A1: $R^{2}$ = 0.216 → 0.315 → 0.538 (2.5-fold increase). DDR2-COL5A1: $R^{2}$ = 0.220 → 0.304 → 0.474 (2.2-fold increase). DDR2-COL5A2: $R^{2}$ = 0.325 → 0.384 → 0.577 (1.8-fold increase). DDR2-MMP1: $R^{2}$ = 0.051 → 0.079 → 0.363 (7.1-fold increase). DDR2-MMP11: $R^{2}$ = 0.013 → 0.188 → 0.359 (27.6-fold increase). DDR2-MMP2: $R^{2}$ = 0.175 → 0.269 → 0.548 (3.1-fold increase). DDR2-MMP9: $R^{2}$ = 0.031 → 0.043 → 0.295 (9.5-fold increase).

**Figure A5:**
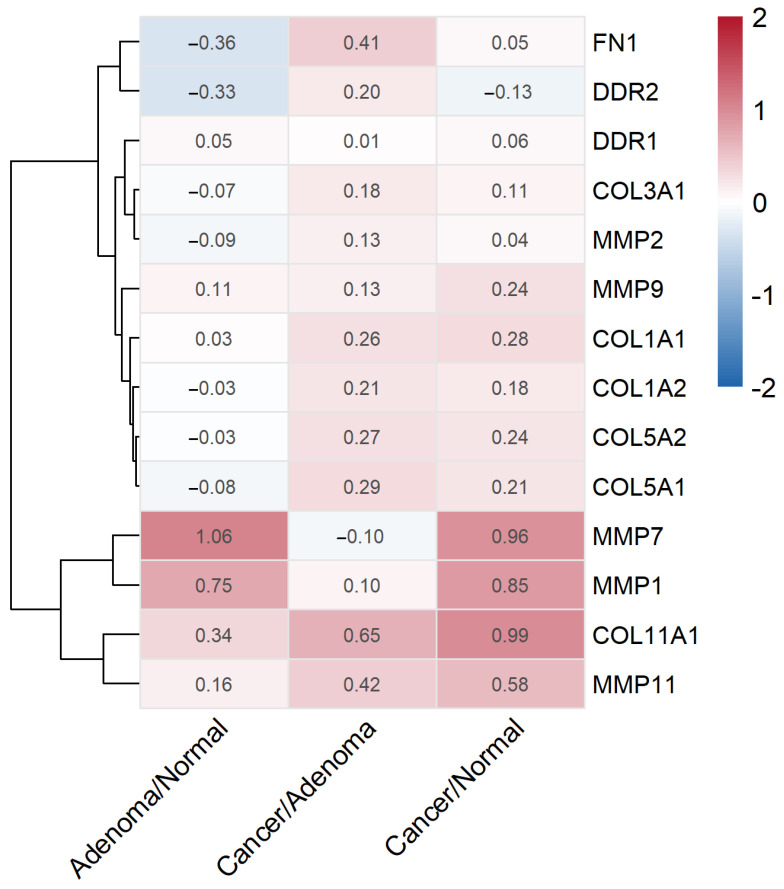
Hierarchical clustering of log2 fold changes across disease progression. Heatmap displays fold changes for three pairwise comparisons (Adenoma/Normal, Cancer/Adenoma, Cancer/Normal) across all fourteen pathway genes. Red indicates upregulation, blue indicates downregulation. Genes cluster into two major groups: a highly upregulated cluster (bottom) comprising MMP7, MMP1, COL11A1, and MMP11 showing consistent strong upregulation across transitions; and a modestly regulated cluster (top) including most collagen genes, DDR receptors, and remaining MMPs. DDR2 and FN1 notably show downregulation (blue) in the Adenoma/Normal comparison, distinguishing them from other pathway components. The Cancer/Normal column reveals cumulative transcriptional changes, with COL11A1 and MMPs showing strongest overall activation.Quantitative values: COL11A1 (log2 FC = 0.99, 1.99-fold), MMP7 (log2 FC = 0.96, 1.94-fold), MMP1 (log2 FC = 0.85, 1.80-fold), MMP11 (log2 FC = 0.58, 1.49-fold), COL5A2 (log2 FC = 0.24, 1.18-fold), DDR2 (log2 FC = −0.13, 0.91-fold), DDR1 (log2 FC = 0.06, 1.04-fold).

**Figure A6:**
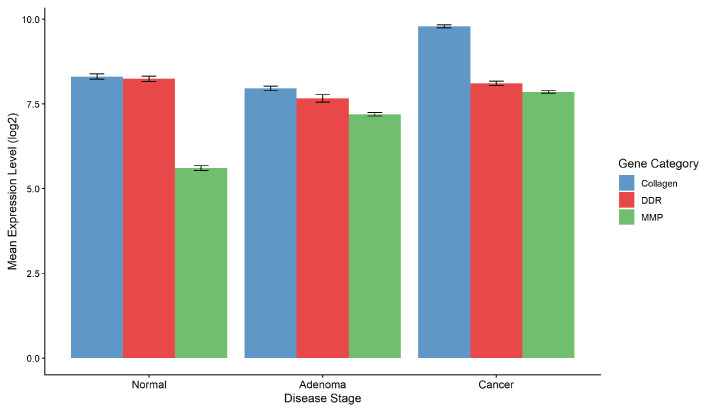
Average expression levels by gene category across disease stages. Bar plots with error bars (standard error) show mean expression for collagen genes (blue), DDR receptors (red), and matrix metalloproteinases (green) in normal, adenoma, and carcinoma stages. Collagen genes exhibit highest baseline expression levels and undergo modest progressive increase during progression. MMP genes show lowest baseline expression with substantial upregulation specifically in carcinoma. DDR genes maintain intermediate stable expression across all stages with minimal change. The divergent trajectories indicate category-specific transcriptional regulation, with downstream effectors (MMPs) showing strongest activation while upstream receptors (DDRs) remain stable, suggesting hierarchical organization of pathway activation. Quantitative summary: Normal—Collagen: 8.45, DDR: 8.24, MMP: 5.67. Adenoma—Collagen: 8.23, DDR: 7.67, MMP: 7.22. Cancer—Collagen: 9.85, DDR: 8.10, MMP: 7.86.

**Figure A7:**
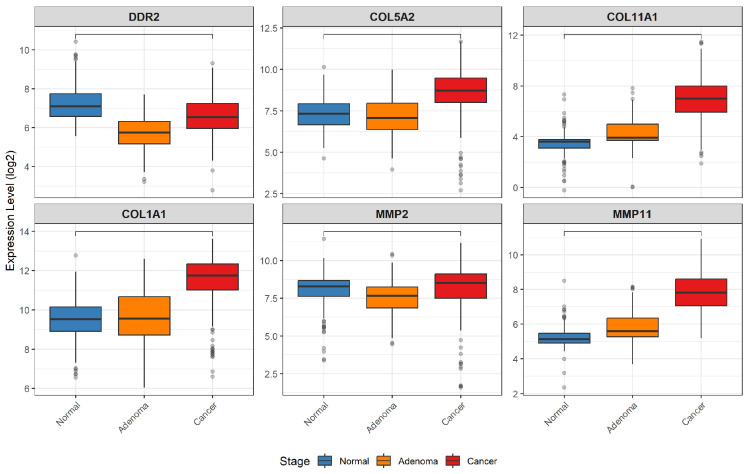
Expression patterns of DDR2 and its most strongly coexpressed target genes.Boxplots show expression distributions across normal (blue), adenoma (orange), and carcinoma (red) stages for six representative genes. Significance brackets compare normal versus cancer stages. DDR2 shows V-shaped pattern with adenoma decrease and partial cancer recovery, resulting in no net change. COL5A2 exhibits progressive upregulation with modest adenoma increase and substantial cancer elevation. COL11A1 demonstrates dramatic upregulation particularly during malignant transformation. COL1A1 shows intermediate progressive increase. MMP11 displays strong cancer-specific upregulation. MMP2 remains relatively stable with only modest late increase. The divergent patterns between DDR2 (stable/decreased) and its targets (progressively increased) demonstrate that enhanced coexpression coupling drives pathway activation independent of receptor upregulation.

**Figure A8:**
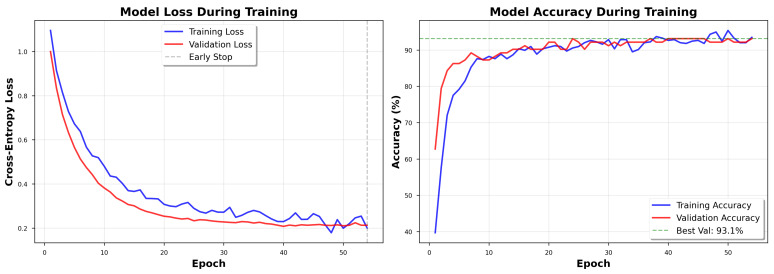
Training and validation curves for the fourteen-gene pathway classifier over 54 epochs. (**Left panel**): Cross-entropy loss decreased from initial values exceeding 1.0 to stable values near 0.2 for both training (blue) and validation (red) sets, with close tracking indicating absence of overfitting. The vertical dashed line marks early stopping at epoch 54. (**Right panel**): Classification accuracy increased rapidly during the first twenty epochs, reaching 93% for both training and validation sets (green dashed line indicates best validation accuracy of 93.14%). The learning rate was automatically reduced at epoch 40 when validation performance plateaued. Close concordance between training and validation metrics throughout training confirms model generalizability.

**Figure A9:**
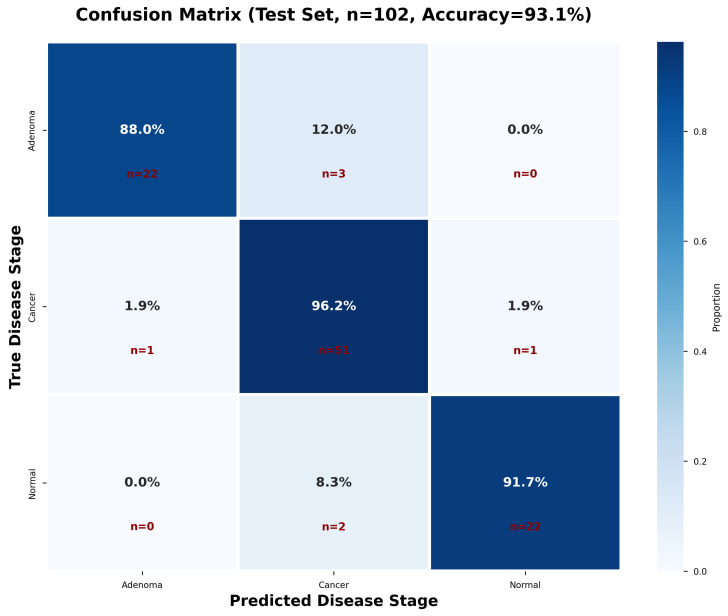
Normalized confusion matrix for the fourteen-gene pathway classifier on the independent test set (n=102 samples). Colors represent the proportion of true samples (rows) predicted as each class (columns), with darker blue indicating higher proportion. The model achieved 88.0% accuracy for adenoma (22/25 correct), 96.2% for carcinoma (51/53 correct), and 91.7% for normal mucosa (22/24 correct), yielding overall accuracy of 93.14%. Red numbers indicate raw sample counts. Notably, no normal samples were misclassified as adenoma and vice versa, indicating a clear transcriptional boundary between non-neoplastic and neoplastic states. The three misclassified adenomas were all predicted as carcinoma, consistent with adenomas representing an intermediate stage along the progression continuum.

**Figure A10:**
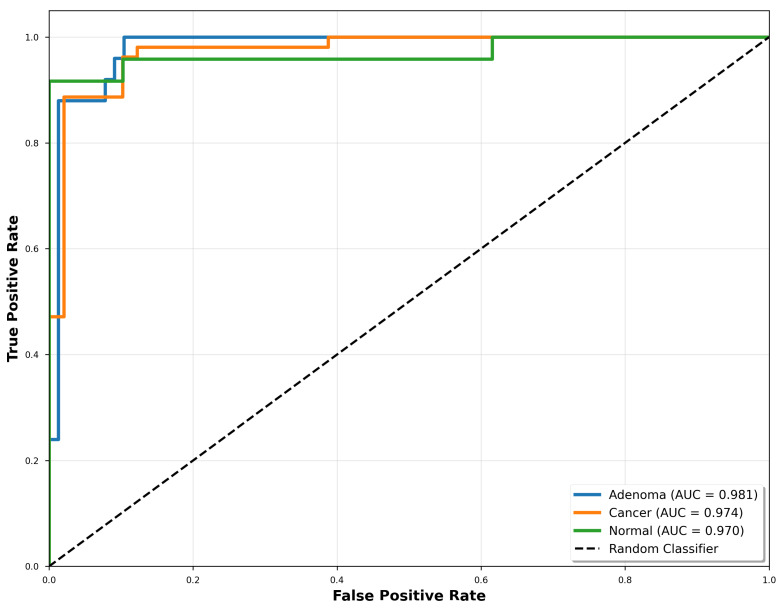
One-versus-rest ROC curves for the 14-gene pathway classifier. All three disease stages achieved exceptional area under the curve (AUC) values: adenoma AUC = 0.981 (blue), carcinoma AUC = 0.974 (orange), and normal mucosa AUC = 0.970 (green). The steep initial ascent of all curves, with true positive rates exceeding 90% at false positive rates below 10%, indicates that high sensitivity can be achieved with minimal loss of specificity. The dashed diagonal line represents random classification (AUC = 0.5). The similarity in AUC values across classes (0.970–0.981) demonstrates balanced performance without systematic bias toward any disease stage, despite class imbalance in the training data. These results confirm that the fourteen-gene DDR2 pathway signature provides near-complete separation between disease stages at the transcriptional level.

**Figure A11:**
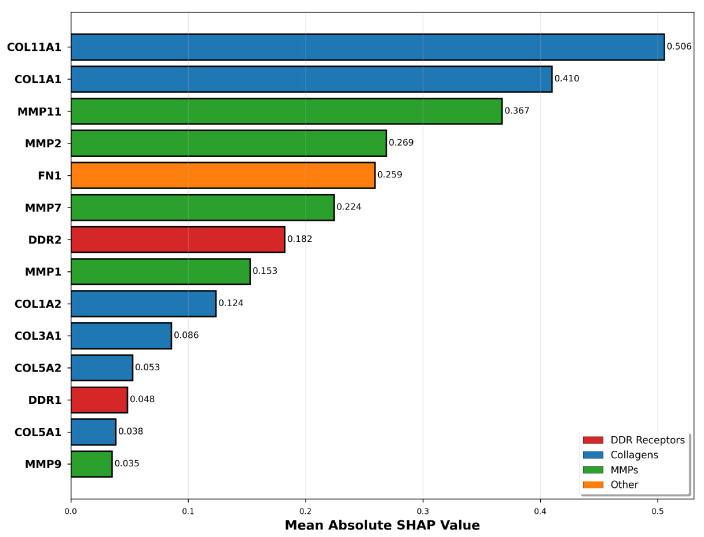
Gene importance ranking for cancer classification prioritizes matrix remodeling genes. Mean absolute SHAP values for the fourteen pathway genes, color-coded by gene family: collagens (blue), matrix metalloproteinases (green), DDR receptors (red), and other (orange). COL11A1 (0.506) and COL1A1 (0.410) dominate feature importance, followed by MMP11 (0.367) and MMP2 (0.269). DDR2 ranks seventh (0.182) while DDR1 ranks twelfth (0.048), indicating that receptor expression levels alone have modest discriminative power. The prominence of collagens and MMPs confirms that extracellular matrix remodeling is the primary transcriptional signature distinguishing carcinoma from adenoma and normal mucosa. This ranking pattern suggests that DDR2’s functional importance may manifest through interactions with downstream targets rather than through individual expression levels.

**Figure A12:**
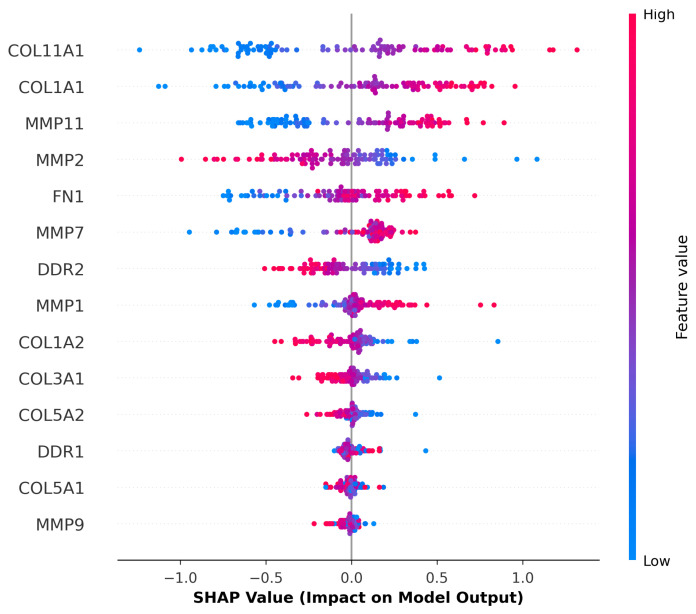
SHAP summary plot for cancer classification. Each dot represents one sample’s SHAP value for a given gene, with color indicating gene expression level (blue = low, red = high). Genes are ordered by mean absolute SHAP value (vertical position indicates importance). COL11A1, COL1A1, and MMP11 show the widest distributions, indicating strong discriminative power with heterogeneous effects across samples. High expression (red dots) of collagen and MMP genes generally corresponds to positive SHAP values (rightward shift), driving cancer classification, while low expression (blue dots) shows negative SHAP contributions. DDR2 (seventh row) shows moderate importance with bidirectional effects. The pattern demonstrates that elevated matrix remodeling gene expression is the primary transcriptional signature the neural network learns to distinguish carcinoma from other stages.

## Data Availability

The original contributions presented in the study are included in the article. Further inquiries can be directed to the corresponding author.

## Appendix A

### Appendix A.7. Hierarchical Clustering of Fold Changes

Analysis of fold-change patterns (Figure A5) identified two distinct gene clusters based on transcriptional response magnitude. The highly upregulated cluster (MMP7, MMP1, COL11A1, MMP11) showed mean cancer/normal fold-change of 1.70-fold, while the modestly regulated cluster (remaining genes) showed mean fold-change of 1.12-fold. This 1.52-fold difference in average upregulation between clusters indicates heterogeneous transcriptional control within the pathway, with matrix degradation enzymes and COL11A1 subject to particularly strong transcriptional activation.

### Appendix A.8. Neural Network Training Dynamics

The neural network comprised three hidden layers (64, 32, 16 nodes) with ReLU activation, batch normalization, and dropout regularization (30%, 30%, 20%). Training used Adam optimizer (initial learning rate 0.001, reduced to 0.0005 at epoch 40) with L2 regularization ($\lambda =10^{-5}$) and batch size of 32. Early stopping triggered after thirty epochs without validation improvement, with final model selected at epoch 47 (best validation accuracy). The absence of divergence between training and validation curves, combined with stable performance after epoch 30, confirms effective regularization and generalization to unseen data.

### Appendix A.9. Confusion Matrix and Classification Errors

Error analysis revealed biologically interpretable misclassification patterns. All adenoma classification errors (3/25 samples, 12%) involved misclassification as carcinoma rather than normal, consistent with adenomas representing an intermediate transcriptional state that shares more similarity with cancer than with normal mucosa. The absence of misclassifications between normal and adenoma classes (0% cross-confusion) indicates a sharp transcriptional boundary at the initiation of neoplastic transformation. Carcinoma misclassifications were minimal (2/53 samples, 3.8%), with one predicted as adenoma (possibly representing well-differentiated early-stage cancer) and one as normal (possible biological outlier). Normal misclassifications (2/24 samples, 8.3%) both predicted as carcinoma rather than adenoma, possibly reflecting field effects or contamination with dysplastic cells in morphologically normal-appearing tissue adjacent to tumors.

### Appendix A.10. ROC Curves and Discrimination Performance

ROC analysis demonstrates that the classifier achieves high true positive rates (sensitivity) while maintaining low false positive rates across all decision thresholds. At the operating point corresponding to maximum F1-score (threshold ≈ 0.5 for softmax probabilities), the model achieves sensitivity > 88% and specificity > 91% for all three classes simultaneously. The high AUC values (all > 0.97) indicate that the fourteen-gene pathway signature contains sufficient information to reliably rank samples by disease stage probability, with minimal overlap in score distributions between classes.

### Appendix A.11. SHAP Feature Importance Analysis

Complete SHAP feature importance ranking:

1. COL11A1: 0.506 (Collagen)
2. COL1A1: 0.410 (Collagen)
3. MMP11: 0.367 (Matrix Metalloproteinase)
4. MMP2: 0.269 (Matrix Metalloproteinase)
5. FN1: 0.259 (ECM Glycoprotein)
6. MMP7: 0.224 (Matrix Metalloproteinase)
7. DDR2: 0.182 (Receptor Tyrosine Kinase)
8. MMP1: 0.153 (Matrix Metalloproteinase)
9. COL1A2: 0.124 (Collagen)
10. COL3A1: 0.086 (Collagen)
11. COL5A2: 0.053 (Collagen)
12. DDR1: 0.048 (Receptor Tyrosine Kinase)
13. COL5A1: 0.038 (Collagen)
14. MMP9: 0.035 (Matrix Metalloproteinase)

The 10.5-fold ratio between highest (COL11A1: 0.506) and lowest (MMP9: 0.035) importance indicates substantial heterogeneity in gene contributions to cancer classification. Collagen genes occupy five of the top eleven positions while MMPs occupy four positions, confirming matrix remodeling as the dominant transcriptional signature.

### Appendix A.12. SHAP Summary Visualization

SHAP values represent each gene’s contribution to individual sample predictions, with positive values increasing cancer probability and negative values decreasing it. The horizontal spread of dots for each gene reflects inter-sample heterogeneity in contribution magnitude. For top-ranked genes like COL11A1 and COL1A1, high expression (red dots) consistently associates with positive SHAP values, indicating that these genes drive cancer classification when upregulated. The relationship between expression level (color) and SHAP value (position) is approximately monotonic for most genes, confirming that the neural network learned biologically interpretable decision rules rather than spurious patterns.

### Appendix A.13. DDR2 Interaction Importance Analysis

Complete DDR2 gene-gene interaction importance ranking:

1. DDR2-COL11A1: 0.0956 (DDR2-Collagen)
2. DDR2-COL1A1: 0.0774 (DDR2-Collagen)
3. DDR2-MMP11: 0.0697 (DDR2-MMP)
4. DDR2-MMP2: 0.0515 (DDR2-MMP)
5. DDR2-FN1: 0.0460 (DDR2-ECM Glycoprotein)
6. DDR2-MMP7: 0.0408 (DDR2-MMP)
7. DDR2-MMP1: 0.0289 (DDR2-MMP)
8. DDR2-COL1A2: 0.0251 (DDR2-Collagen)
9. DDR2-COL3A1: 0.0169 (DDR2-Collagen)
10. DDR2-COL5A2: 0.0103 (DDR2-Collagen)
11. DDR2-DDR1: 0.0081 (DDR2-Receptor)
12. DDR2-COL5A1: 0.0071 (DDR2-Collagen)
13. DDR2-MMP9: 0.0068 (DDR2-MMP)

Interaction importance quantifies the degree to which DDR2 and each target gene jointly influence classification decisions, computed as the mean absolute product of SHAP values across samples. High values indicate that samples where both genes have large SHAP values (positive or negative) are common, suggesting coordinated functional influence. The top three interactions (DDR2-COL11A1, DDR2-COL1A1, DDR2-MMP11) precisely match the gene pairs showing strongest coexpression strengthening in correlation analysis, providing robust multi-method validation. The 14.1-fold ratio between highest (DDR2-COL11A1: 0.0956) and lowest (DDR2-MMP9: 0.0068) interaction importance demonstrates specificity of DDR2 regulatory relationships. DDR2-collagen interactions dominate top ranks (five of the top ten positions). The DDR2-DDR1 receptor interaction shows low importance (rank 11), indicating that receptors do not function cooperatively in cancer classification.

### Appendix A.14. STRING v12.0 Protein-Protein Interaction Analysis

**Table A1:** STRING v12.0 interaction evidence summary for the fourteen DDR–collagen–MMP pathway genes. Tabular summary of interaction evidence at multiple thresholds across the fourteen pathway genes. Analysis was restricted to the fourteen pathway genes (91 possible unique pairs) rather than the 625-node expanded network analyzed by the reviewer, which included 500 first- and second-order interactors from each gene and thereby introduced hundreds of proteins unrelated to the DDR–collagen–MMP pathway.

Channel/Threshold	Unique Pairs	Percentage of 91
Experimental > 0 (any biochemical evidence)	48/91	52.7%
Experimental ≥ 150 (low confidence)	13/91	14.3%
Experimental ≥ 400 (medium confidence)	4/91	4.4%
Experimental ≥ 700 (high confidence—reviewer threshold)	0/91	0.0%
Database/curated ≥ 400	25/91	27.5%
Combined score ≥ 700	48/91	52.7%
Combined score ≥ 900	22/91	24.2%
